# SERRATE Regulates Floral Meristem Activity by Antagonizing SHOOT MERISTEMLESS and Repressing Cytokinin Signaling

**DOI:** 10.1002/advs.75540

**Published:** 2026-05-07

**Authors:** Wen Yang, Yiting Wang, Yuxin You, Zhiyue Wu, Dongbao Li, Xin Wang, Yongsheng Chang, Hang Zhao, Tao Zhu, Dijun Chen, Wei Chen, Bo Sun

**Affiliations:** ^1^ State Key Laboratory of Pharmaceutical Biotechnology School of Life Sciences Nanjing University Nanjing China

**Keywords:** cytokinin, floral meristem, KNAT2, miRNA, SE, STM

## Abstract

*SHOOT MERISTEMLESS* (*STM*), a homeobox transcription factor, functions to maintain shoot apical meristem (SAM) and floral meristem (FM) activity. Carpel formation is abolished in *stm* mutant flowers, but this phenotype can be partially restored by compromising the function of SERRATE (SE), the core component in miRNA formation. However, whether and how SE functions in meristem maintenance and flower development remains mysterious. Here, we show that the partial loss‐of‐function mutant of *SE* (*se‐1*) shows additional floral organs and enlarged SAM sizes, and could restore FM activity in the *stm* mutant. We further demonstrate that SE represses the expression of STM‐targeted *KNOTTED‐LIKE FROM ARABIDOPSIS THALIANA 2* (*KNAT2*) through miR171c‐5p, thereby inhibiting the expression of *ISOPENTENYL TRANSFERASE 7* (*IPT7*), which is activated by STM and is required for cytokinin biosynthesis. *IPT7* can also be repressed by SE through the miR164c–CUP‐SHAPED COTYLEDON 1/2 (CUC1/2)–KNAT2 regulatory module. Thus, FM activity promoted by restored cytokinin signaling could restore carpel formation in the *se stm* double mutant. The antagonistic regulation between SE and STM for FM maintenance ensures proper carpel development in Arabidopsis.

## Graphical abstract

Floral meristem (FM) activity is crucial for the initiation of floral organs. In this study, SE represses *IPT7* expression and cytokinin signaling via both the miR171c‐5p–KNAT2 and miR164c–CUC1/2–KNAT2 regulatory modules, thereby repressing FM activity. In contrast, STM induces both *KNAT2* and *IPT7* expression. The antagonistic roles between SE and STM ensure proper FM homeostasis and flower development.

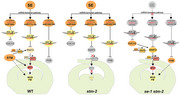

## 1 Introduction

The nuclear zinc‐finger protein SERRATE (SE) plays crucial roles in plant development, and its close homologs are widely found in plants, fungi, and animals [1, 2, 3]. In plants, SE has been well known to collaborate with Dicer‐like 1 (DCL1) and Hyponastic Leaves 1 (HYL1) to precisely process primary miRNAs (pri‐miRNAs) into precursor miRNAs (pre‐miRNAs) and subsequently into miRNA/miRNA* duplexes during miRNA maturation [4, 5, 6, 7, 8]. Loss‐of‐function of *SE* (*se‐1*) leads to a global reduction in mature miRNAs and pleiotropically perturbs plant development, resulting in phenotypes including abnormal embryogenesis, delayed leaf initiation, accelerated phase change, serrated and curled leaves, and aberrant phyllotaxy in the inflorescence [2, 9, 10, 11]. However, the detailed function of SE‐mediated miRNAs in flower development remains largely unknown.

Floral meristem (FM) activity within floral buds, which are produced from the peripheral zone of the shoot apical meristem (SAM), is crucial for the initiation of floral organs. In *Arabidopsis thaliana*, the homeobox transcription factor (TF) *WUSCHEL* (*WUS*), which is expressed in the organizing center (OC), functions in maintaining the stem cell pools of both SAM and FM [12]. From the OC, WUS migrates upward through the plasmodesmata to the central zone (CZ) and activates the expression of the stem cell marker gene *CLAVATA3* (*CLV3*) [13], whereas the CLV3 peptide diffuses from the stem cells to the OC and restricts *WUS* expression [14, 15]. Thus, the *CLV–WUS* negative feedback loop ensures the homeostasis of both SAM and FM [16].

Phytohormones, especially auxin and cytokinin, have been reported to play key roles in regulating meristematic activity [17, 18, 19, 20, 21]. Auxin is biosynthesized by YUCCAs (YUCs) and transported by PIN‐FORMEDs (PINs) to accumulate at the incipient primordium sites to form auxin maxima, which promote cell differentiation and organogenesis through auxin response factors (ARFs) [22, 23, 24, 25, 26]. On the other hand, OC represents a cytokinin‐accumulating zone, and cytokinin activates *WUS* expression in the OC and maintains meristematic activity [27, 28].

Class I *knotted‐like homeobox* (*KNOX*) genes are differentially required for meristem maintenance and reproductive organogenesis [29]. The Arabidopsis genome contains four Class I *KNOX* genes: *SHOOT MERISTEMLESS* (*STM*), *KNOTTED‐LIKE FROM ARABIDOPSIS THALIANA 2* (*KNAT2*), *BREVIPEDICELLUS* (*BP* or *KNAT1*), and *KNAT6* [30]. *KNAT2* expresses in the vegetative apical meristem and may function during carpel development [31, 32]. In dexamethasone (DEX)‐induced *35S:KNAT2‐GR* plants, ectopic carpels and carpel‐like structures are formed [31]. STM is also specifically expressed in meristematic regions [33, 34], and can robustly and rapidly activate the expression of cytokinin biosynthesis genes, including *ISOPENTENYL TRANSFERASE 7* (*IPT7*), thereby promoting cytokinin signaling to sustain meristem activity [35, 36]. Weakened *STM* function prevents SAM formation and disrupts floral organ development, with carpels being notably absent in flowers of the weak *stm‐2* allele [9, 33, 37]. Notably, loss‐of‐function of *SE* partially restores the absence of pistils in *stm‐2* flowers [9], suggesting an antagonistic role between SE and STM in carpel development. However, whether and how SE participates in FM regulation remain poorly understood.

In this article, we have outlined the function of *SE* in FM regulation. The *se‐1* mutant showed additional floral organs and enlarged SAM sizes. SE could act through the dual miR171c‐5p–KNAT2 and miR164c–CUP‐SHAPED COTYLEDON 1/2 (CUC1/2)–KNAT2 pathways to repress cytokinin signaling, thereby weakening FM activity. In contrast, through CUT&Tag assays, we identified global STM downstream targets in flower development and showed that STM activated *KNAT2* expression for FM maintenance. Therefore, introducing the *se* allele into the *stm* background could partially restore the abolished FM activity and the absence of pistils in *stm‐2*. Overall, the antagonistic regulation of FM activity between *SE* and *STM* ensures proper carpel development in Arabidopsis.

## 2 Results

### 2.1 SE Regulates FM Activity by Repressing Cytokinin Signaling

**FIGURE 1:**
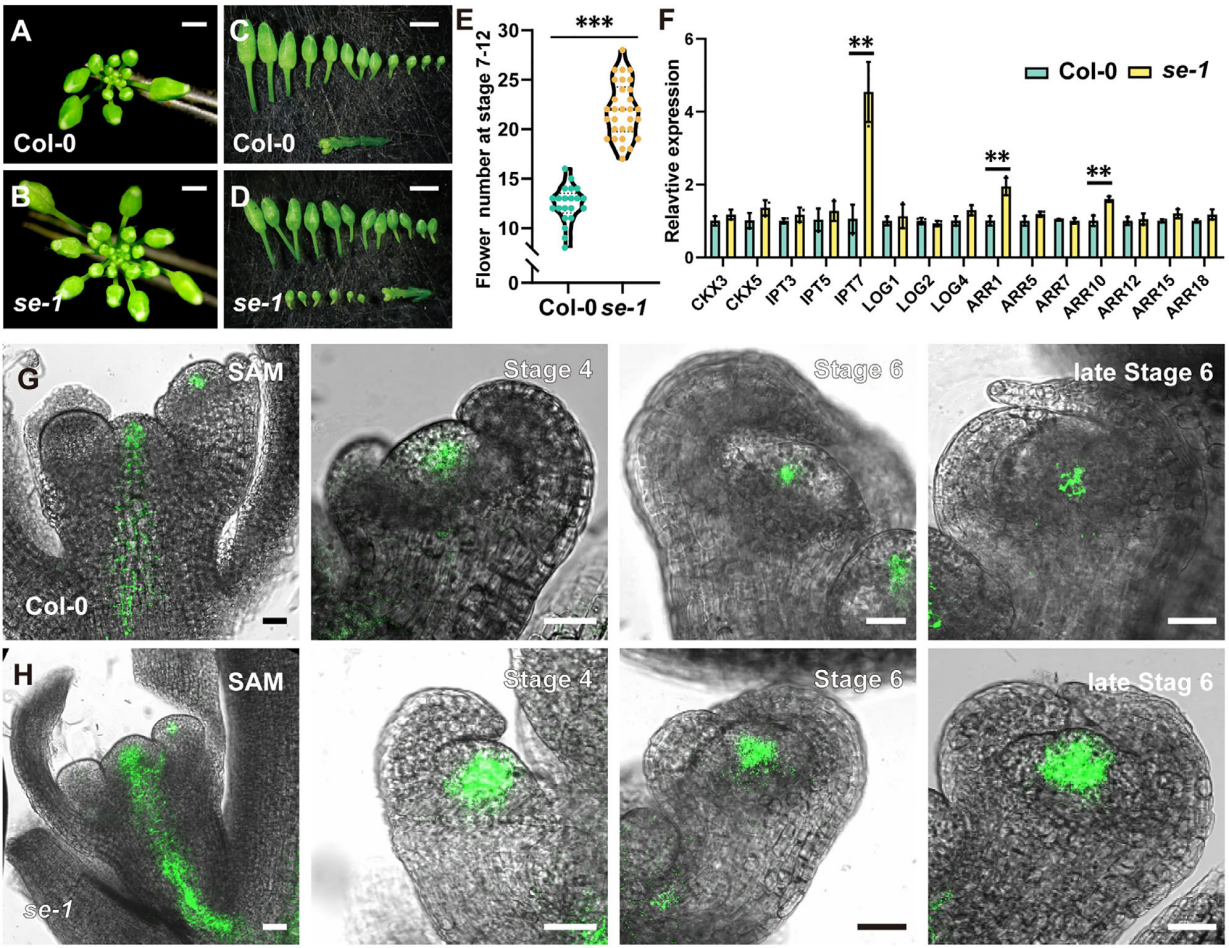
SE regulates FM activity by repressing cytokinin signaling. (A, B) Inflorescence phenotypes of wild‐type (Col‐0) (A) and *se‐1* (B). Scale bar, 1 mm. (C, D) Representation of total flowers per inflorescence in Col‐0 (C) and *se‐1* (D). Scale bars, 2 cm. (E) Statistical analysis of the number of stage 7∼12 flowers per inflorescence in Col‐0 and *se‐1* (n = 30). Flowers of 30 randomly picked inflorescences from 30 independent plants (1 inflorescence per plant) from three independent repetitions (10 plants per repetition) were used for analysis. (F) RT‐qPCR analysis of *CKXs* (*CKX3* and *CKX5*), *IPTs* (*IPT3*, *IPT5* and *IPT7*), *LOGs* (*LOG1*, *LOG2*, and *LOG4*), and B‐type *ARRs* (*ARR1*, *ARR5*, *ARR7*, *ARR10*, *ARR12*, *ARR15*, and *ARR18*) expression levels in Col‐0 and *se‐1* inflorescence. (G, H) Confocal observation of ProTCSn:GFP signals in SAM, stage 4, stage 6 and late stage 6 floral buds of Col‐0 (G) and *se‐1* (H). Scale bars, 20 µm. The significant differences are calculated using Student's *t‐test* for (E) and two‐way ANOVA followed by Tukey's multiple comparison test for (F). Statistically significant differences are indicated by *p<0.05, **p<0.01, and ***p<0.001. Independent biological replicates (with three technical replicates each) in (F): n = 3. Values are means ± SD.

*SE* mutation leads to pleiotropic developmental phenotypes [9, 38]. To test whether *SE* participates in flower development, we examined the partial loss‐of‐function mutant *se‐1*. In *se‐1* flowers, an increased number of petals (28.57%) and the presence of additional filamentous‐like structures (4.76%) were observed (Figure S1A–D,F). Additionally, the number of flowers at floral stages 7∼12 per inflorescence in *se‐1* was significantly higher than that in the wild‐type (Figure 1A–E). We also transformed *e* *GFP*‐tagged *SE* driven by the *SE* native promoter into *se‐1*. The abnormal flower phenotypes of *se‐1* were fully rescued in *se‐1 pSE:SE‐eGFP* plants (Figure S1E–H), confirming that the mutation in *SE* is responsible for the floral defects. Furthermore, the pistil‐less phenotype of *stm‐2* was partially restored upon introduction of *se‐1* into the *stm‐2* background (Figure S1I–K), resulting in 1∼2 unfused carpels in the fourth floral whorl, as previously reported [9]. The unfused carpels contained differentiated stigmatic tissues at their distal apices, and possibly due to aberrant or incomplete valve marginal fusion, distinctive protrusions were observed along the valve margins (Figure S1I–K). In *se‐1 stm‐2*, the unfused carpels were possibly caused by the loss of STM function, which was specifically expressed in the replum of the silique (Figure S1L) [39]. As reported [33, 40], *STM* was also expressed in the SAM (Figure S1M). Interestingly, randomly formed SAMs were observed on the shoots of *se‐1 stm‐2* plants (Figure S2). Together, these results indicate that the loss of *SE* function promotes both SAM and FM activity.

STM has been reported to control meristem activity by regulating hormones, particularly cytokinins [36]. Based on the partially restored FM and carpels in *se‐1 stm‐2* floral buds (Figure S1I–K), we examined auxin and cytokinin signaling outputs in *se‐1* floral buds by introducing the *DR5:GFP‐ER* [41] and *ProTCSn:GFP* [20] reporters, respectively. In the SAM and floral buds at different stages of wild‐type plants, DR5:GFP‐ER signals, which indicate auxin signaling output, were primarily detected at the lateral organ initiation sites within the SAM and at the tips of the sepals within floral buds. This pattern was similar in *se‐1 DR5:GFP‐ER* plants (Figure S3A,B). Consistently, the transcription levels of several *PIN* (*PIN1*, *PIN3*, *PIN4*, and *PIN7*) genes and *YUC* (*YUC1*, *YUC2*, and *YUC4*) genes, which may function during floral development [42, 43], were unchanged in *se‐1* compared to those in the wild‐type (Figure S3C). However, ProTCSn:GFP signals, which represent cytokinin signaling output and are located in the OC beneath the stem cells (Figure 1G,H), increased dramatically in the SAM and FMs of stage 4 and 6 floral buds in *se‐1 ProTCSn:GFP* compared to those in *ProTCSn:GFP* (Figure 1G,H).

**FIGURE 2:**
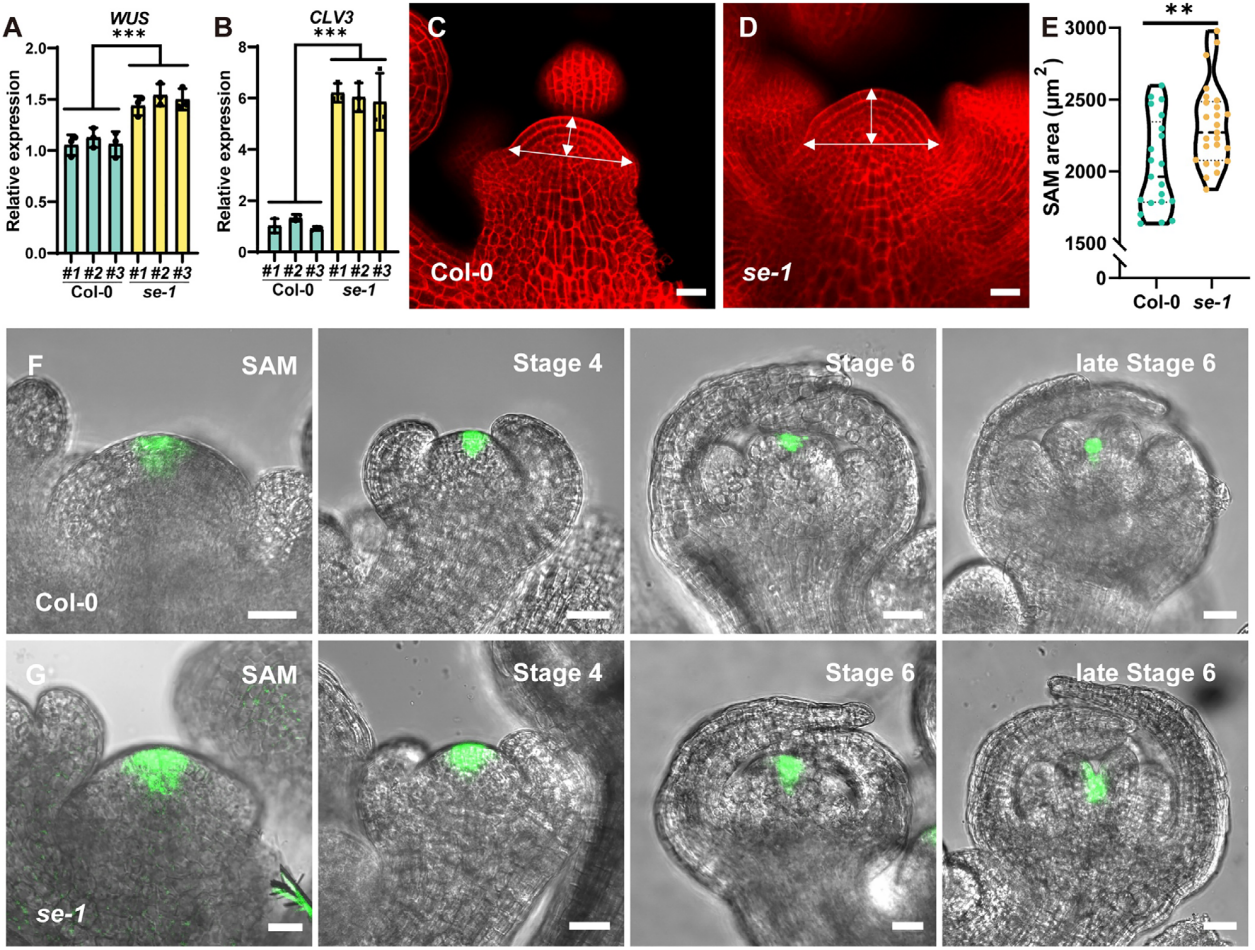
SE represses WUS and CLV3 expression and SAM sizes. (A, B) RT‐qPCR analysis of *WUS* (A) and *CLV3* (B) expression levels in Col‐0 and *se‐1* inflorescences. (C, D) Confocal observation of Col‐0 (C) and *se‐1* (D) SAMs. Bidirectional white arrows indicate diameter and height, respectively. SAMs were stained with Propidium Iodide (PI). Scale bars, 20 µm. (E) Statistical analysis of SAM areas of Col‐0 and *se‐1* (n = 30). SAMs of 30 randomly picked inflorescences from 30 independent plants (1 inflorescence per plant) from three independent repetitions (10 plants per repetition) were used for analysis. (F, G) Confocal observation of *pCLV3:GFP‐ER* signals in SAM, stage 4, stage 6 and late stage 6 floral buds of WT (F) and *se‐1* (G) plants. Scale bars, 20 µm. The significant differences are calculated using two‐way ANOVA followed by Tukey's multiple comparison test for (A) and (B), and Student's *t‐test* for (E). Statistically significant differences are indicated by **p<0.01 and ***p<0.001. Independent biological replicates (with three technical replicates each) in (A) and (B): n = 3. Values are means ± SD.

In Arabidopsis, IPTs catalyze the synthesis of cytokinin; LONELY GUYs (LOGs) facilitate the production of active cytokinin; CYTOKININ OXIDASEs (CKXs) promote the degradation of cytokinin [44, 45]; and B‐type ARABIDOPSIS RESPONSE REGULATORs (ARRs) act as transcriptional activators in the cytokinin signaling pathway [46]. To identify potential cytokinin‐related targets of SE, we performed real‐time quantitative reverse transcription PCR (RT‐qPCR) and found that the expression of *IPT7*, *ARR1*, and *ARR10* was upregulated in *se‐1* compared to the wild‐type, with *IPT7* showing the most pronounced increase, while the expression of other tested cytokinin‐related genes showed no significant differences (Figure 1F). Cytokinin has been reported to activate *WUS* expression for meristem development [28], and B‐type ARR1, ARR10, and ARR12 also redundantly promote *WUS* transcription [18, 47, 48, 49, 50]. In turn, WUS activates the stem cell marker *CLV3* [13]. Consistently, RT‐qPCR showed that both *WUS* and *CLV3* expression were significantly upregulated in *se‐1* floral buds compared to those in the wild‐type (Figure 2A,B), consistent with the increased cytokinin activity in *se‐1* (Figure 1F–H). Moreover, the *se‐1* mutant showed enlarged SAM sizes compared to the wild‐type (Figure 2C–E; Figure S4), as previously reported for *se‐2* and *se‐3* [9]. We also introduced the *pCLV3:GFP‐ER* reporter into *se‐1* [51], and the GFP signals in *se‐1 pCLV3:GFP‐ER* significantly increased compared with those in *pCLV3:GFP‐ER* (Figure 2F,G). These results suggest that SE could act through cytokinin signaling for both SAM and FM activity regulation.

### 2.2 KNAT2 is the Key Target of SE for FM Activity Regulation

**FIGURE 3:**
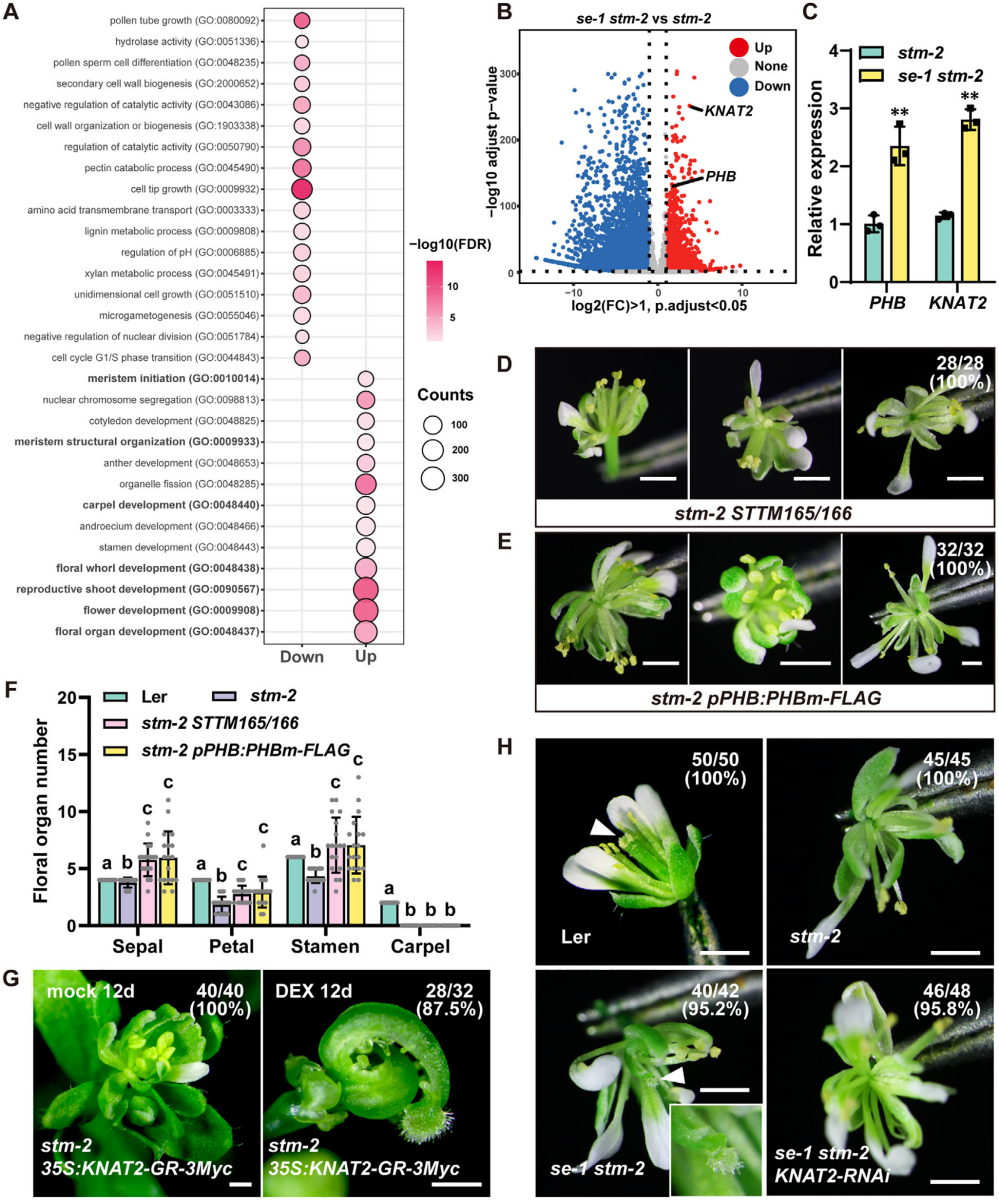
KNAT2 is the key target of SE‐mediated pathway for FM activity regulation. (A) Gene Ontology analysis of the downregulated and upregulated genes in early inflorescences of *se‐1 stm‐2* compared to those in *stm‐2*. (B) Differentially expressed genes in early inflorescences of *se‐1 stm‐2* compared with those in *stm‐2* by RNA‐Seq. (C) RT‐qPCR analysis of *PHB* and *KNAT2* expression levels in *stm‐2* and *se‐1 stm‐2* floral buds. (D, E) Flower phenotypes of *stm‐2 STTM165/166* (D, n = 28) and *stm‐2 pPHB:PHBm‐FLAG* (E, n = 32). Randomly picked flowers from 10 independent plants (2∼4 flowers per plant) from 3 independent lines (3∼4 plants per line) were used for analysis. Scale bars, 1 mm. (F) Statistical analysis of floral organ numbers in Ler, *stm‐2*, *stm‐2 STTM165/166*, and *stm‐2 pPHB:PHBm‐FLAG* (n = 20). Floral organs of 20 randomly picked flowers from 10 independent plants (2 flowers per plant) from two independent repetitions (10 plants per repetition) were used for analysis. Different letters (a, b, and c) indicate significant differences between groups (*p<0.05). (G) Flower phenotypes of *stm‐2 35S:KNAT2‐GR‐3Myc* 12 d after mock (left, n = 40) or DEX (right, n = 32) treatment. Randomly picked flowers from 20 independent plants (1∼2 flowers per plant) from 3 independent lines (6∼7 plants per line) were used for analysis. Scale bars, 500 µm. (H) Flower phenotypes of Ler (n = 50), *stm‐2* (n = 45), *se‐1 stm‐2* (n = 42), and *se‐1 stm‐2 KNAT2‐RNAi* (n = 48). Randomly picked flowers from 20 independent plants (2∼3 flowers per plant) from two independent repetitions (10 plants per repetition) were used for analysis. The white arrowhead indicates the pistil. The inset is the close‐up view of the carpel‐like structure. Upper right numbers in (D, E) and (G, H) show the phenotype frequency (individuals with phenotype/total, %). Scale bars, 1 mm. The significant differences are calculated using two‐way ANOVA followed by Tukey's multiple comparison test for (C) and (F). Statistically significant differences in (C) are indicated by **p<0.01. Independent biological replicates (with three technical replicates each) in (C): n = 3. Values are means ± SD.

SE typically functions in the processing of miRNAs to regulate downstream plant development [4, 5, 6]. Because *IPT7* is not a reported miRNA target, we then analyzed the transcriptomes of *stm‐2* and *se‐1 stm‐2* floral buds (≤ stage 8) by RNA‐seq with two biological replicates to identify the potential targets of SE‐mediated miRNA pathway in flower development (Figure S5A,B; Dataset S1). In *se‐1 stm‐2* floral buds, we detected 1,593 (fold change > 1.5, *p* < 0.05) upregulated and 4,236 (fold change > 1.5, *p* < 0.05) downregulated genes relative to *stm‐2* (Dataset S2). The gene ontology (GO) analysis of differentially expressed genes in *se‐1 stm‐2* floral buds showed that upregulated genes were enriched in categories related to meristem initiation, carpel development, and floral whorl development, whereas significant enrichment in GO categories of downregulated genes was related to cell tip growth, pectin catabolic process, and pollen tube growth (Figure 3A; Dataset S3).

Given the repressive role of SE on miRNA‐targeted genes, the downregulated genes in *se‐1 stm‐2* should be indirect; therefore, we focused on the upregulated genes, which included *PHABULOSA* (*PHB*) and *KNAT2* (Figure 3B; Dataset S2). The SE‐miR165/166 target *PHB* could regulate FM activity [9, 52, 53, 54], and *KNAT2* is a homolog of *STM* in the *KNOX* family [30]. The increased expression levels of *PHB* and *KNAT2* in *se‐1 stm‐2* were further confirmed by RT‐qPCR (Figure 3C). To test whether the regenerated pistils in *se‐1 stm‐2* were caused by upregulated *PHB*, we crossed *stm‐2* with *pPHB:PHBm‐FLAG*, which expresses a form of *PHB* resistant to miR165/166 cleavage, and with *Short Tandem Target Mimic 165/166* (*STTM165/166*) [55, 56, 57], an artificial miR165/166 target mimic that degrades miR165/166 and reduces miR165/166 activities, respectively [58]. While the average numbers of sepals, petals, and stamens increased in *stm‐2 STTM165/166* and *stm‐2 pPHB:PHBm‐FLAG* flowers compared to those in the wild‐type and *stm‐2*, no pistil initiation was observed in these two lines (Figure 3D–F). These results indicate that although SE can regulate FM function by repressing *PHB* expression via the miRNA pathway, *PHB* is not the key target responsible for the partial restoration of pistils in the *se‐1 stm‐2* background.

We then focused on *KNAT2*. RT‐qPCR showed that *KNAT2* was significantly upregulated in *se‐1* and *se‐1 stm‐2* floral buds, and downregulated in *stm‐2* compared to the wild‐type (Figure S5C,D), while the expression of other class‐I *KNOX* family members, including *BP* and *KNAT6*, was comparable across the wild‐type and different mutant lines (Figure S5E), suggesting that *KNAT2* is possibly the specific downstream target of SE within this family. We then generated *35S:KNAT2‐GR‐3Myc* and *stm‐2 35S:KNAT2‐GR‐3Myc* lines in which *KNAT2* coding sequence (CDS) was fused with the steroid‐binding domain of the rat glucocorticoid receptor (GR), thereby conferring inducible *KNAT2* activity upon DEX treatment (Figure S5F,G) [59, 60]. Compared with the mock‐treated *stm‐2 35S:KNAT2‐GR‐3Myc* flowers, which lacked pistils, continuous DEX treatment for 12 days largely restored the carpel defects in *stm‐2*, resulting in unfused carpels (Figure 3G) resembling those of *se‐1 stm‐2* (Figure S1K) [9]. Moreover, the knockdown of *KNAT2* expression in *se‐1 stm‐2* abolished pistil formation, and the plants reverted to the *stm‐2* phenotype (Figure 3H). Therefore, *KNAT2* acts as the key target of SE for carpel development in the *stm‐2* background.

### 2.3 SE Represses KNAT2 Expression Through miR171c‐5p

**FIGURE 4:**
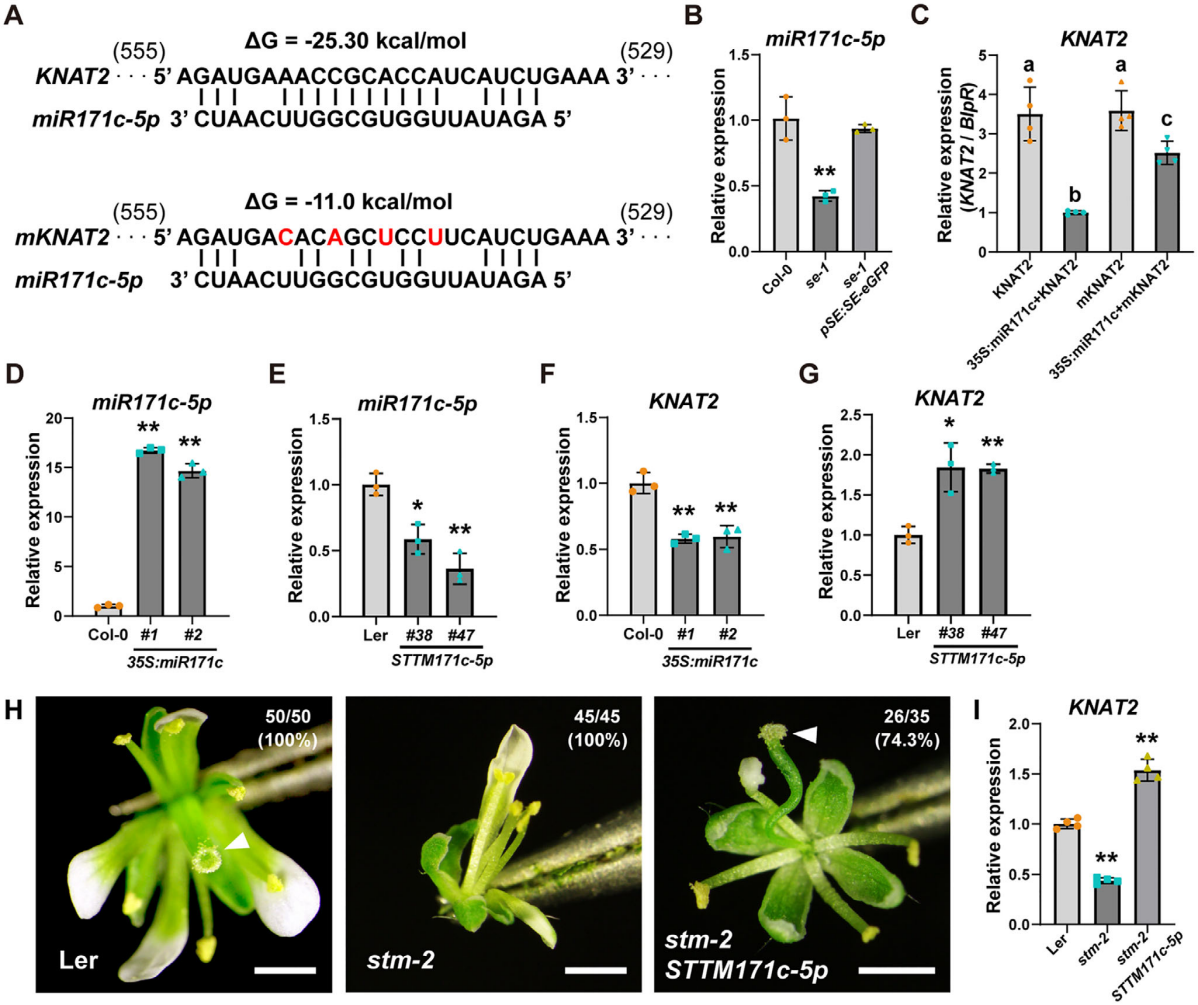
SE inhibits KNAT2 expression via miR171c‐5p. (A) Alignment of partial mRNA sequences of *KNAT2* and miR171c‐5p. *mKNAT2* is the modified mRNA that harbors synonymous nucleotide substitutions (bases in red) in *miR171c‐5p* binding site. Free energies of duplex structures were calculated using the Mfold method [61]. (B) RT‐qPCR analysis of *miR171c‐5p* expression levels in Col‐0, *se‐1*, and *se‐1 pSE:SE‐eGFP* floral buds. (C) RT‐qPCR analysis of *KNAT2* and *mKNAT2* expression levels in different co‐infiltration groups in tobacco leaves. Different letters (a, b, and c) indicate significant differences between groups (*p<0.05). (D‐G) RT‐qPCR analysis of *miR171c‐5p* expression levels in *35S:miR171c* (D) and *STTM171c‐5p* (E) floral buds, and *KNAT2* expression levels in *35S:miR171c* (F) and *STTM171c‐5p* (G). (H) Flower phenotypes of Ler (n = 50), *stm‐2* (n = 45), and *stm‐2 STTM171c‐5p* (n = 35). Randomly picked flowers from 20 independent plants (1∼3 flowers per plant) from two independent repetitions (10 plants per repetition) were used for analysis. The white arrowheads indicate the pistils. Upper right numbers show the phenotype frequency (individuals with phenotype/total, %). Scale bars, 1 mm. (I) RT‐qPCR analysis of *KNAT2* expression levels in Ler, *stm‐2*, and *stm‐2 STTM171c‐5p* floral buds. The significant differences are calculated using one‐way ANOVA followed by Tukey's multiple comparison test for (B‐G) and (I). Statistically significant differences in (B), (D‐G), and (I) are indicated by *p<0.05 and **p<0.01. Independent biological replicates (with three technical replicates each): n = 3. Values are means ± SD.

To test whether *KNAT2* is a target of miRNAs, we performed sequence alignment using the miRbase database with *KNAT2* mRNA. A mature miRNA, named miR171c‐5p (Accession number: MIMAT0031900), was identified at a pairing rate of 17/21 (paired nucleotides/the total targeted nucleotides) (Figure 4A) [61]. The abundance of mature miR171c‐5p was noticeably reduced in *se‐1* floral buds compared to the wild‐type and was restored to wild‐type levels in the complemented *se‐1 pSE:SE‐eGFP* line, indicating that the biogenesis of miR171c‐5p depends on SE (Figure 4B). To determine whether *KNAT2* is targeted by miR171c‐5p, we prepared constructs expressing either the wild‐type *KNAT2* or a mutant version (*mKNAT2*) driven by the native *KNAT2* promoter. The *mKNAT2* variant encodes the same amino acid sequence as wild‐type *KNAT2* but produces a transcript that is resistant to miR171c‐5p cleavage (Figure 4A) [61]. When co‐expressed with constitutively expressed *miR171c‐5p* (*35S:miR171c*) in *Nicotiana benthamiana* (*N. benthamiana*) leaves, the *mKNAT2* expression level in the *35S:miR171c* plus *pKNAT2:mKNAT2* group was higher than that of *KNAT2* in the *35S:miR171c* plus *pKNAT2:KNAT2* group (Figure 4C). Additionally, *35S:miR171c* and *STTM171c‐5p* transgenic Arabidopsis were also generated (Figure 4D,E) [55, 56, 57]. *KNAT2* expression was significantly downregulated in *35S:miR171c* floral buds but upregulated in *STTM171c‐5p* floral buds compared to that in the wild‐type (Figure 4F,G). Thus, *KNAT2* is a direct target of miR171c‐5p.

We then introduced *STTM171c‐5p* into the *stm‐2* background. The absence of pistils in *stm‐2* was also partially restored in *stm‐2 STTM171c‐5p* (Figure 4H), similar to the carpel phenotypes of *se‐1 stm‐2* and *stm‐2 35S:KNAT2‐GR‐3Myc* with induced *KNAT2* overexpression (Figure 3G,4H; Figure S1K). Additionally, the *KNAT2* expression was higher in *stm‐2 STTM171c‐5p* than in *stm‐2* (Figure 4I). Together with the evidence for the direct cleavage of *KNAT2* by miR171c‐5p (Figure 4A–G), these results suggest that SE represses *KNAT2* expression via miR171c‐5p to regulate FM activity.

### 2.4 SE–miR171c‐5p–KNAT2 Module Regulates FM Activity Through Repressing IPT7 Expression

**FIGURE 5:**
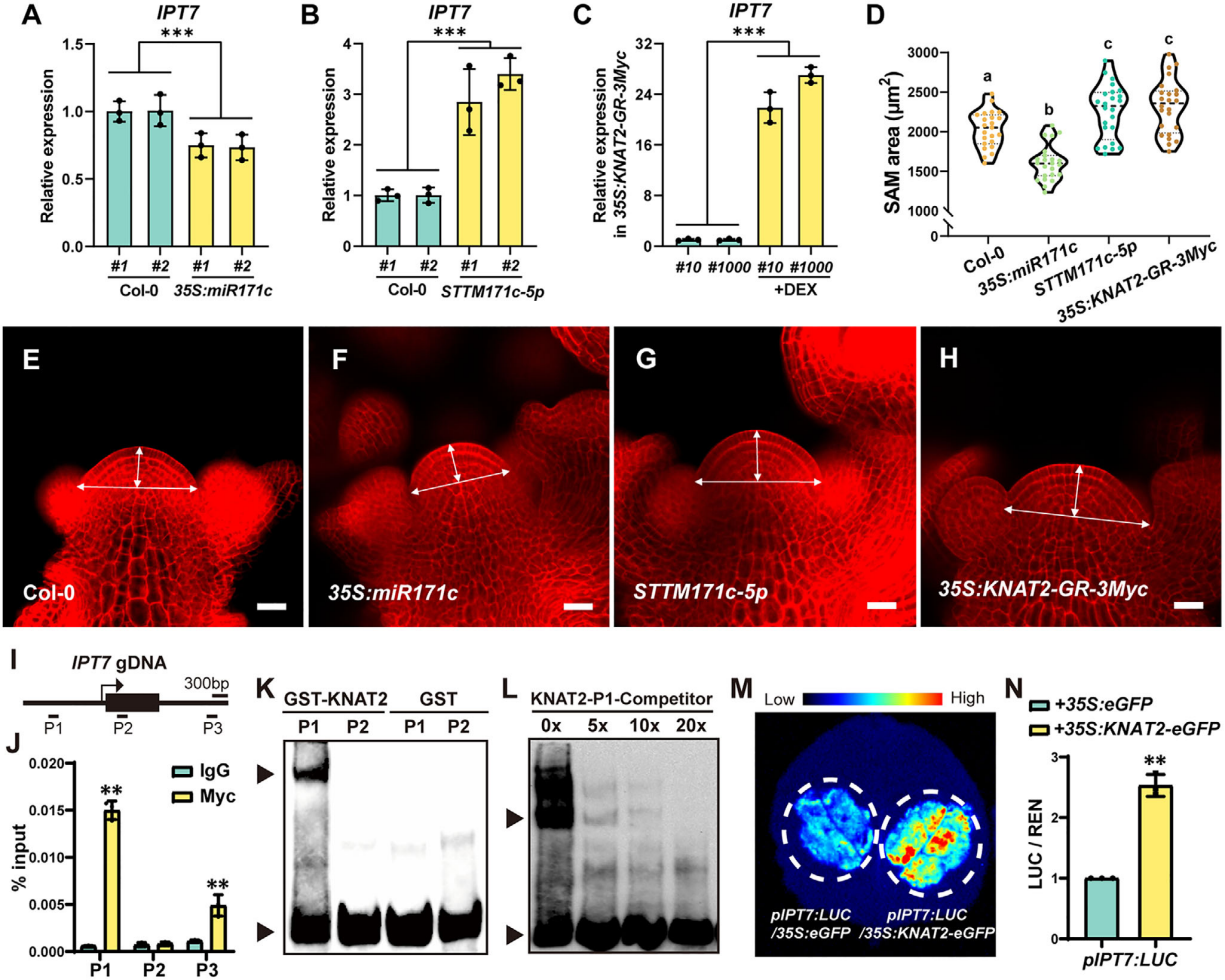
SE–miR171c‐5p–KNAT2 module regulates FM activity by repressing IPT7 expression. (A‐C) RT‐qPCR analysis of *IPT7* expression levels in Col‐0, *35S:miR171c* (A), *STTM171c‐5p* (B), and DEX‐induced *35S:KNAT2‐GR‐3Myc* (C) floral buds. (D) Statistical analysis of SAM areas of Col‐0, *35S:miR171c*, *STTM171c‐5p*, and DEX‐induced *35S:KNAT2‐GR‐3Myc* (n = 25). SAMs of 25 randomly picked inflorescences from 25 independent plants (1 inflorescence per plant) from two independent repetitions (12∼13 plants per repetition) were used for analysis. Different letters (a, b, and c) indicate significant differences between groups (*p<0.05). (E‐H) Confocal observation of Col‐0 (E), *35S:miR171c* (F), *STTM171c‐5p* (G) and DEX‐induced *35S:KNAT2‐GR‐3Myc* (H) SAMs. Bidirectional white arrows indicate diameter and height, respectively. SAMs were stained with PI. Scale bars, 20 µm. (I) Schematic representation of the gene structure of *IPT7*. The black box represents the exon. P1, P2 and P3 indicate the regions chosen for analysis. (J) Binding of KNAT2 to the *IPT7* locus in floral buds of *35S:KNAT2‐GR‐3Myc*. P1, P2 and P3 indicate the regions chosen for ChIP‐qPCR. The y‐axis shows the calibrated relative ratio of bound DNA to input DNA after IP, and IgG served as the negative control. P1, P2 and P3 regions are shown in (I). (K, L) EMSA between GST‐KNAT2 and the putative KNAT2‐binding fragment P1 of the *IPT7* promoter with (L) or without the competitor (K). The black arrowheads indicate the protein‐DNA complexes (upper) and free probes (lower). Only GST and the P2 region were used as the negative controls in (K). The unlabeled P1 was used as the competitor in gradient concentrations in (L). P1 and P2 regions are shown in (I). (M) Comparison of dual‐luciferase assays between *pIPT7:LUC* plus *35S:KNAT2‐eGFP* and *pIPT7:LUC* plus *35S:eGFP* in tobacco leaves. At least 15 infiltrated leaves from three independent biological replicates (at least 5 leaves per replicate) were used for analysis. (N) Relative luciferase intensity in (M) was quantitatively presented by RT‐qPCR. *REN* was used as the internal control. The significant differences are calculated using two‐way ANOVA followed by Tukey's multiple comparison test for (A‐C) and (J), one‐way ANOVA followed by Tukey's multiple comparison test for (D), and Student's *t‐test* for (N). Statistically significant differences in (A‐C), (J), and (N) are indicated by **p<0.01 and ***p<0.001. Independent biological replicates (with three technical replicates each): n = 3. Values are means ± SD.

STM has been reported to control SAM activity by promoting cytokinin signaling [36]. As *KNAT2* is a homolog of *STM* and *IPT7* expression was elevated in *se‐1* flowers (Figure 1F) [30], we wondered whether the SE–miR171c‐5p–KNAT2 module regulates FM activity through *IPT7*. Thus, we examined *IPT7* expression in *35S:miR171c*, *STTM171c‐5p*, and *35S:KNAT2‐GR‐3Myc* floral buds. Compared with the wild‐type or mock group, *IPT7* expression was downregulated in *35S:miR171c* and upregulated in both *STTM171c‐5p* and DEX‐treated *35S:KNAT2‐GR‐3Myc* (Figure 5A–C), similar to the expression change of *IPT7* in *se‐1* (Figure 1F). Consistently, the changes in SAM size observed in the *35S:miR171c*, *STTM171c‐5p*, and *35S:KNAT2‐GR‐3Myc* lines were broadly associated with altered *IPT7* expression levels, although the relationship was not strictly proportional (Figure 5D–H), similar to the expression changes of *WUS* and *CLV3* (Figure S6). Additionally, the treatment of *se‐1 stm‐2* floral buds with the cytokinin biosynthesis inhibitor lovastatin abolished the formation of unfused carpels (Figure S7). These findings suggest that the SE–miR171c‐5p–KNAT2 module acts through cytokinin for FM activity regulation.

We also tested the binding of KNAT2 to the *IPT7* genomic locus. ChIP‐qPCR showed that KNAT2 directly bound to the *IPT7* promoter and the region downstream of its transcription end site (TES), with strong enrichment at the promoter (P1) (Figure 5I,J). This specific promoter binding was further confirmed by electrophoretic mobility shift assays (EMSAs) and luciferase (LUC) assays (Figure 5K–N). Combined with the changed expression of *IPT7* in *se‐1*, *35S:miR171c*, *STTM171c‐5p*, and *35S:KNAT2‐GR‐3Myc* (Figures 1F and 5A–C), these results indicate that the SE–miR171c‐5p–KNAT2 module could repress FM activity through cytokinin signaling by directly downregulating *IPT7* expression.

### 2.5 SE–miR164c–CUC1/2 Module Could Repress FM Activity Through downregulating KNAT2 Expression

**FIGURE 6:**
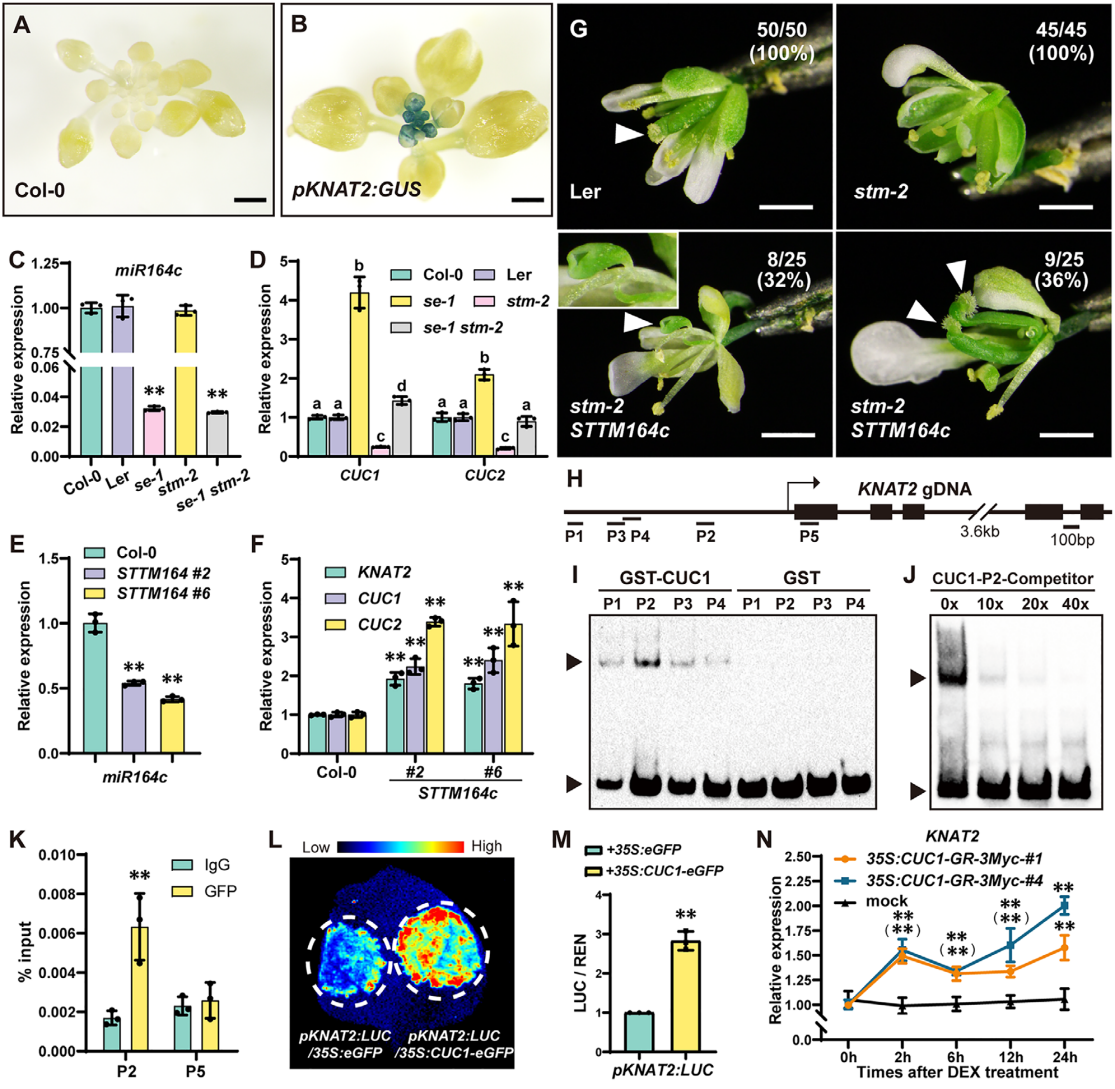
SE–miR164c–CUC1/2 module could repress FM activity by regulating KNAT2 expression. (A, B) Observation of *pKNAT2:GUS* signals in the Col‐0 (A) and *pKNAT2:GUS* (B) inflorescences. Scale bars, 500 µm. (C, D) RT‐qPCR analysis of *miR164c* (C) and *CUC1* and *CUC2* (D) expression levels in Col‐0, Ler, *se‐1*, *stm‐2*, and *se‐1 stm‐2* flowers. Different letters (a, b, c, and d) indicate significant differences between groups (*p<0.05). (E, F) RT‐qPCR analysis of *miR164c* (E) and *KNAT2*, *CUC1*, and *CUC2* (F) expression levels in Col‐0 and *STTM164c* floral buds. (G) Flower phenotypes of Ler (n = 50), *stm‐2* (n = 45), and *stm‐2 STTM164c* (n = 25). Randomly picked flowers from 10∼20 independent plants (2∼3 flowers per plant) from two independent repetitions (5∼10 plants per repetition) were used for analysis. The white arrowheads indicate the pistils. The inset is the close‐up view of the carpel‐like structure. Upper right numbers show the phenotype frequency (individuals with phenotype/total, %). Scale bars, 1 mm. (H) Schematic representation of the gene structure of *KNAT2*. The black boxes represent the exons. P1, P2, P3, P4, and P5 indicate the regions chosen for analysis. (I, J) EMSA between GST‐CUC1 and the selected fragments (P1, P2, P3, and P4) of *KNAT2* promoter with (J) or without the competitor (I). The black arrowheads indicate the protein‐DNA complexes (upper) and free probes (lower). Only GST was used as the negative control in (I). The unlabeled P2 was used as the competitor in gradient concentrations in (J). P1‐P4 regions are shown in (H). (K) Binding of CUC1 to the *KNAT2* locus in floral buds of *35S:CUC1‐GR‐3Myc*. P2 and P5 indicate the regions chosen for ChIP‐qPCR. The y‐axis shows the calibrated relative ratio of bound DNA to input DNA after IP, and IgG served as the negative control. P2 and P5 regions are shown in (H). (L) Comparison of dual‐luciferase assays between *pKNAT2:LUC* plus *35S:CUC1‐eGFP* and *pKNAT2:LUC* plus *35S:eGFP* in tobacco leaves. At least 15 infiltrated leaves from three independent biological replicates (at least 5 leaves per replicate) were used for analysis. (M) Relative luciferase intensity in (L) was quantitatively presented by RT‐qPCR. *REN* was used as the internal control. (N) RT‐qPCR analysis of *KNAT2* expression levels at different time points after DEX treatment in *35S:CUC1‐GR‐3Myc* floral buds. The significant differences are calculated using one‐way ANOVA followed by Tukey's multiple comparison test for (C) and (E), two‐way ANOVA followed by Tukey's multiple comparison test for (D), (F), (K), and (N), and Student's *t‐test* for (M). Statistically significant differences in (C), (E‐F), (K), (M) and (N) are indicated by **p<0.01. Independent biological replicates (with three technical replicates each): n = 3. Values are means ± SD.

The miR164c‐mediated *CUC1* and *CUC2* genes encode NAC domain proteins that are highly conserved relative to the petunia NO APICAL MERISTEM protein [62, 63, 64, 65], and the *cuc1‐1 cuc2‐1* double mutant completely lacks embryonic SAM [66]. Moreover, *CUC1* directly promotes *STM* expression [64, 67], and the expression patterns of *STM* and *KNAT2* largely overlap in regions that have been reported to also express *CUC1* and *CUC2* (Figure 6A,B; Figure S1M) [68, 69]. These observations prompted us to examine whether the regenerated pistils in *se‐1 stm‐2* were also involved in the miR164c–CUC1/2 pathway, which may potentially regulate the *STM* homolog *KNAT2*. The level of mature miR164c was obviously reduced in *se‐1* and *se‐1 stm‐2* floral buds compared to that in the wild‐type and *stm‐2* (Figure 6C). Conversely, the expression of both *CUC1* and *CUC2*, targets of miR164c, was elevated in *se‐1* (compared with the wild‐type) and *se‐1 stm‐2* (compared with *stm‐2*) (Figure 6D). We then generated *STTM164c* transgenic Arabidopsis to sequester miR164c (Figure 6E). *CUC1* and *CUC2* expression was significantly upregulated in *STTM164c* floral buds compared to that in the wild‐type (Figure 6F), further demonstrating that *CUC1/2* are direct targets of miR164c [65]. Moreover, in transgenic Arabidopsis expressing *35S:STM‐GR‐3Myc*, *35S:KNAT2‐GR‐3Myc*, and *35S:CUC1‐GR‐3Myc*, we observed similar floral abnormalities, especially in the overgrown pistils (Figure S8). Consistently, *KNAT2* was also upregulated in *STTM164c* floral buds (Figure 6F), similar to the *35S:CUC1* plants [66], implying that *KNAT2*, like *STM*, may be a target of CUC1/2.

Subsequently, we introduced *STTM164c* into the *stm‐2* background. The absence of pistils in *stm‐2* was partially restored in *stm‐2 STTM164c* flowers (Figure 6G), suggesting that the miR164c targets CUC1/2 are also crucial for carpel development in the *stm‐2* background. To test the binding ability of CUC1/2 to the *KNAT2* genomic locus, we chose CUC1 to perform EMSA assays and found that CUC1 directly bound to the *KNAT2* promoter, especially in the P2 region (Figure 6H–J). This binding was further confirmed in vivo by ChIP‐qPCR and LUC assays (Figure 6K–M). In addition, *KNAT2* expression was markedly induced in *35S:CUC1‐GR‐3Myc* floral buds after continuous DEX treatment (Figure 6N). Combined with the upregulated expression of *KNAT2* in *se‐1* and *STTM164c* (Figure 6F; Figure S5C), these results demonstrate that the SE–miR164c–CUC1/2 module could control *KNAT2* expression in the *stm‐2* background for FM regulation.

To test whether SE regulates FM activity through miR171c‐5p and miR164c, we generated the *stm‐2 STTM171c‐5p STTM164c* Arabidopsis. The absence of pistils in *stm‐2* was obviously restored in *stm‐2 STTM171c‐5p STTM164c* (Figure S9A–C), with a restoration rate of 81.6%. This rate was higher than that in *stm‐2 STTM171c‐5p* (74.3%) and *stm‐2 STTM164c* (68%) and comparable to that in the DEX‐treated *stm‐2 35S:KNAT2‐GR‐3Myc* (87.5%) (Figure S9D), indicating that the regulation of FM activity by SE is largely dependent on both miR171c‐5p and miR164c.

### 2.6 STM Directly Activates KNAT2 Expression

**FIGURE 7:**
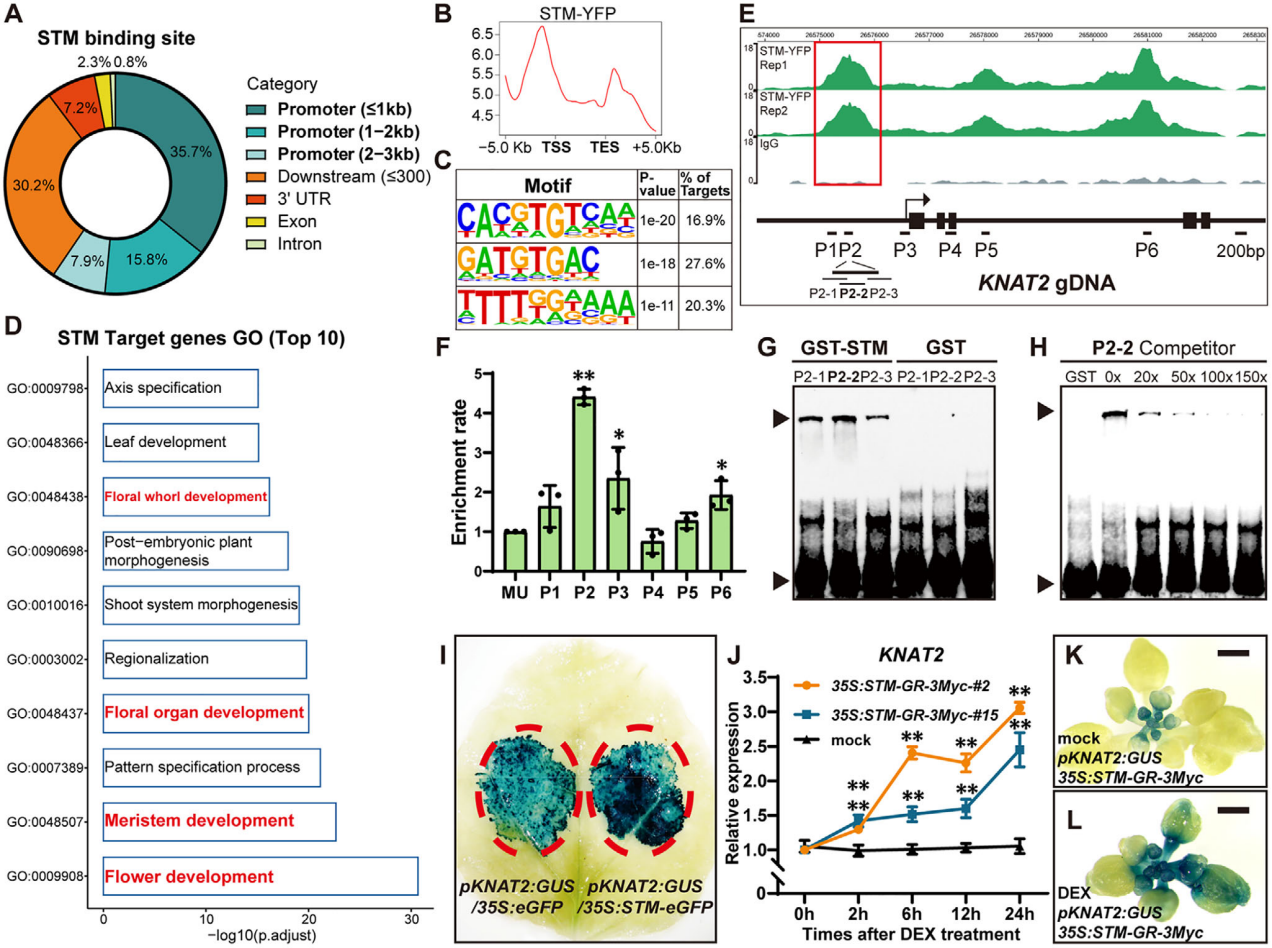
Global binding of STM in SAM and floral buds, as well as STM binding to KNAT2 for its activation. (A) Pie chart representation of the distribution of STM peaks identified by CUT&Tag in different genomic regions. (B) Density plot showing the average enrichment of STM around all genes. CPM‐normalized CUT&Tag densities of equal bins (bin = 50 bp) along the gene and 5 kb region flanking the TSS and TES were plotted. (C) The top three binding motifs of STM on its targets. (D) Gene Ontology analysis of STM‐binding genes. (E) CUT&Tag of STM enrichment on the *KNAT2* locus in *stm‐11 pSTM:STM‐YFP* floral buds (upper) with schematic representation of *KNAT2* gene structure (below). The black boxes represent the exons. P1, P2, P3, P4, P5, and P6 indicate the regions chosen for analysis, and P2 could be divided into three fragments (P2‐1, P2‐2, and P2‐3). (F) Binding of STM to the *KNAT2* locus in floral buds of *stm‐11 pSTM:STM‐YFP*. P1‐P6 indicate the regions chosen for ChIP‐qPCR. The Mu‐like transposon (MU) served as a negative control and the MU values were normalized to 1. P1‐P6 regions are shown in (E). (G, H) EMSA between GST‐STM and the selected fragments (P2‐1, P2‐2, and P2‐3) of *KNAT2* promoter with (H) or without the competitor (G). The black arrowheads indicate the protein‐DNA complexes (upper) and free probes (lower). Only GST was used as the negative control in (G). The unlabeled P2‐2 was used as the competitor in gradient concentrations in (H). P2‐1, P2‐2, and P2‐3 regions are shown in (E). (I) Comparison of GUS signals between *pKNAT2*:*GUS* plus *35S*:*GFP* and *pKNAT2*:*GUS* plus *35S:STM‐GFP* in tobacco leaves. At least 12 infiltrated leaves from three independent biological replicates (at least 4 leaves per replicate) were used for analysis. (J) RT‐qPCR analysis of *KNAT2* expression levels at different time points after DEX treatment in *35S:STM‐GR‐3Myc* floral buds. (K, L) Observation of GUS signals in *pKNAT2*:*GUS 35S:STM‐GR‐3Myc* inflorescences after mock (K) and DEX treatment (L). At least 15 inflorescences from 15 independent plants (1 inflorescence per plant) from three independent lines (at least 5 plants per line) were used for analysis. Scale bars, 500 µm. The significant differences are calculated using one‐way ANOVA followed by Tukey's multiple comparison test for (F), and two‐way ANOVA followed by Tukey's multiple comparison test for (J). Statistically significant differences in (F) and (J) are indicated by *p<0.05 and **p<0.01. Independent biological replicates (with three technical replicates each): n = 3. Values are means ± SD.

It has been reported that STM can induce *KNAT2* expression [70, 71]. Consistently, *KNAT2* expression was reduced in the *stm‐2* floral buds (Figure S5D). To test whether the STM global targets include *KNAT2* during flower development, we performed Cleavage Under Targets & Tagmentation (CUT&Tag) assays for global STM‐YFP binding in *stm‐11 pSTM:STM‐YFP* floral buds [69] and identified 802 STM binding genes (Dataset S1 and S4). After peak annotation, we analyzed STM binding in the Arabidopsis genome and observed a significant accumulation of STM binding on regions upstream of transcription start sites (TSSs, ≤3 kb) (Figure 7A). The STM enrichment profile consistently showed a major peak near the TSS and also a minor peak near the TES (Figure 7B). De novo motif discovery within these bound regions identified a dominant (C/G)A(C/T)GTG(T/A)C G‐box‐like motif [72], which was found in 44.5% of all STM targets (Figure 7C). This motif is similar to the putative CTGTCA STM‐binding site in *CUC1* [68]. The analysis of GO terms showed that STM‐binding genes were enriched in categories related to flower, meristem, and floral organ development (Figure 7D). Additionally, STM also directly bound to the *KNAT2* locus, particularly to the promoter region (Figure 7E). This was confirmed by ChIP‐qPCR and EMSA (Figure 7E–H). We then co‐expressed the *pKNAT2:GUS* reporter with *35S:eGFP* or *35S:STM‐eGFP* in *N. benthamiana* leaves. The GUS signals in the presence of *35S:STM‐eGFP* were stronger than those in the *pKNAT2:GUS* plus *35S:eGFP* group (Figure 7I). Additionally, *KNAT2* expression was markedly induced after the continuous DEX treatment of *35S:STM‐GR‐3Myc* floral buds (Figure 7J). The induction of *KNAT2* expression by STM was also confirmed in *pKNAT2:GUS 35S:STM‐GR‐3Myc* Arabidopsis (Figure 7K,L; Figure S10). Thus, STM directly binds to the *KNAT2* promoter and activates *KNAT2* expression.

**FIGURE 8:**
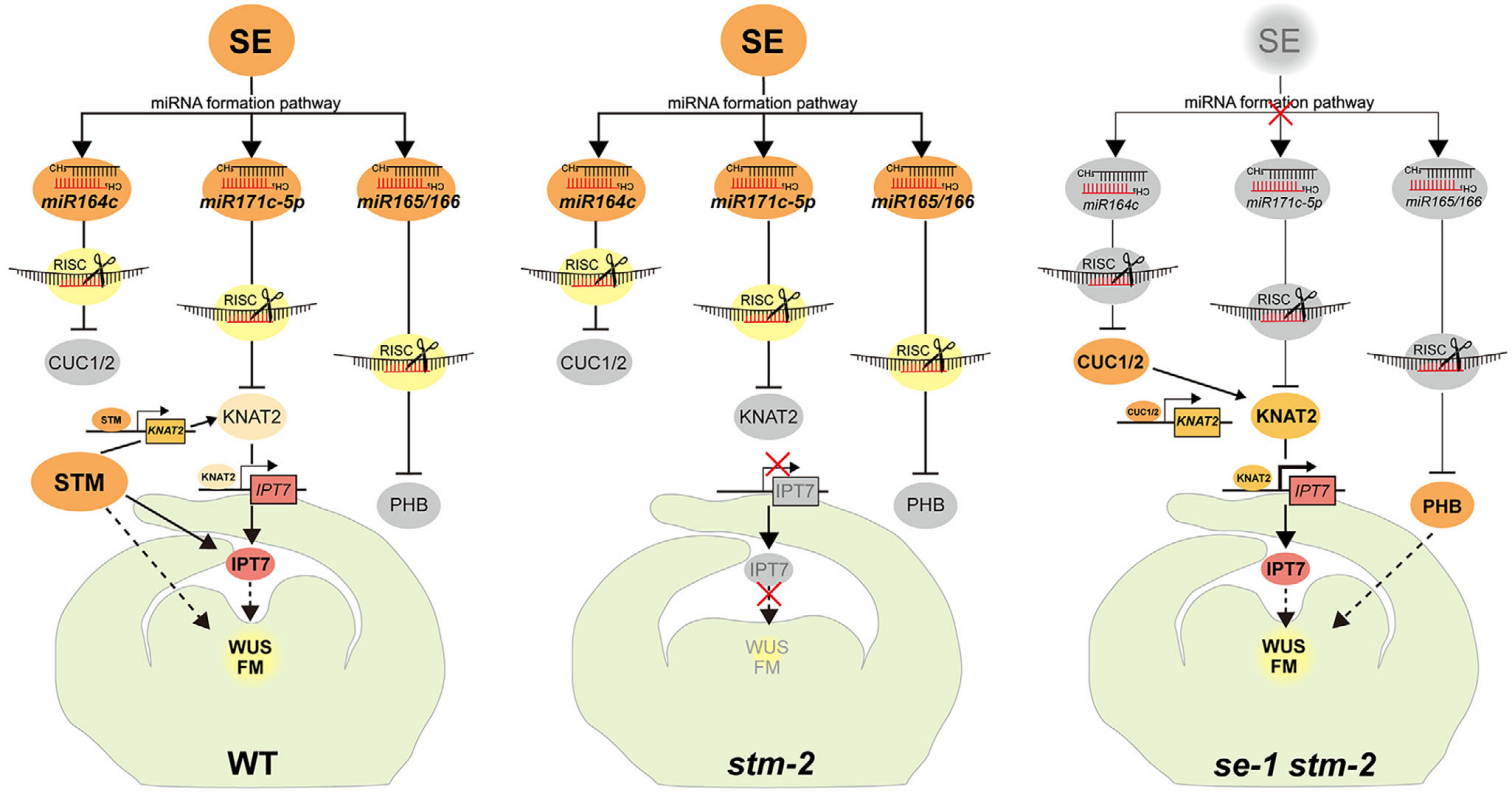
SE and STM antagonistically regulate FM activity through cytokinin signaling. In wild‐type, STM ensures proper FM function through cytokinin signaling. In *stm‐2*, cytokinin activity is largely weakened due to loss of STM function and the repression of *IPT7* expression via SE‐mediated miRNA pathway. In *se‐1 stm‐2*, loss‐of‐function of *SE* induces *IPT7* expression via miR171c‐5p–KNAT2 and miR164c–CUC1/2–KNAT2 modules, thereby re‐activating cytokinin signaling and ultimately restoring FM activity in *stm‐2* background. Besides, mutating *SE* could also elevate *PHB* expression, which results in increased floral organs. The antagonistic regulation between SE and STM ensures proper FM activity.

Generally, our study demonstrates that SE plays an essential role in FM regulation (Figure 8). SE represses *IPT7* expression and cytokinin signaling via both miR171c‐5p–KNAT2 and miR164c–CUC1/2–KNAT2 regulatory modules, thereby repressing FM activity. In contrast, STM induces both *KNAT2* and *IPT7* expression. The antagonistic role between SE and STM ensure proper FM homeostasis and flower development.

## 3 Discussion

The establishment and maintenance of FM requires precise regulatory networks involving multiple factors [59]. In this study, we found that *SE* could regulate FM activity. Loss‐of‐function of *SE* resulted in elevated *CLV3* expression (Figure 2B,F,G) and increased cytokinin activity (Figure 1F–H), resulting in enlarged SAM sizes (Figure 2C–E; Figure S4) and more flowers per inflorescence than in the wild‐type (Figure 1A–E). Furthermore, *se‐1 stm‐2* showed partially restored pistils compared to the absence of *stm‐2* (Figure S1I–K) [9]. This regulation of restored FM activity and carpel formation requires specific miRNAs—miR171c‐5p and miR164c which are dependent on SE. While miR171c‐5p directly cleaved *KNAT2* mRNA for downstream *IPT7* regulation (Figures 4, 5A–C, and 5I–N), miR164c acted through *CUC1/2* (Figure 6C–F) [65], which can bind to the *KNAT2* promoter for *KNAT2* induction (Figure 6H–N). Thus, along with traditional master transcriptional regulators, including *AG* and *KNU* [73, 74, 75, 76], SE‐mediated miRNAs are another crucial type of factors that repress FM activity and regulate flower development. Other well‐known examples include *APETALA2* (*AP2*) and the Class III HD‐ZIP transcription factors *PHB* and *PHAVOLUTA* (*PHV*). *AP2* and several *AP2*‐like genes harbor miR172 binding sites. Ectopic expression of miR172 throughout flowers leads to defects in floral organ identity resembling those of strong *ap2* mutants, with abnormalities in the first‐whorl sepals and second‐whorl petals [21, 77]. In addition, miR165/166 and their targets (*PHB* and *PHV*) also influence FM activity and termination [58, 78, 79]. However, given the vast number of miRNAs in plants, the functions of other miRNAs in flower development remain to be explored.

Unlike the *stm‐2* mutant, which lacks pistils in flowers [9, 33, 37], *KNAT2* mutation does not affect flower development [80]. However, the carpel defects in *stm‐2* were largely restored in *stm‐2 35S:KNAT2‐GR‐3Myc* after DEX treatment (Figure 3G). In *Prunus mume*, *PmKNAT2* is also crucial for pistil number regulation [81]. In addition, overexpression of the *STM* homolog *BP* can restore carpel development in the *stm‐1* mutant [70]. These findings suggest that *STM*, *BP*, *KNAT2*, and possibly other *STM* homologs, may have similar functions in the regulation of FM activity. Because STM can directly activate *KNAT2* expression (Figure 7E–L), the loss of *STM* function weakens KNAT2 activity. This may explain why *KNAT2* fail to regulate FM activity in the *stm‐2* background in a compensatory manner. However, whether other *STM* homologs can regulate FM activity remains to be investigated, as they are not the targets of SE‐mediated miRNAs (Figure S5E; Dataset S2).

STM is specifically expressed in meristematic regions and could robustly and rapidly activate the expression of *IPT7*, thereby promoting cytokinin signaling to sustain meristem activity [35, 36]. Consistent with this, introducing *pSTM:IPT7* into the *stm* mutant largely restored the absence of SAM in *stm* [36]. In the present study, both STM and the SE‐mediated miRNA pathway converged on *IPT7* to regulate FM activity (Figure 8). Furthermore, in agreement with a previous report [9], the *se‐1* mutant exhibited enlarged SAM sizes compared to the wild‐type (Figure 2C–E; Figure S4), and ectopically formed SAMs were observed in the *se‐1 stm‐2* double mutant (Figure S2). Given that STM and SE share *IPT7* as a common downstream target and that SE functions in SAM, these results suggest that the SE‐mediated miRNA pathway may also contribute to the regulation of SAM activity in addition to its role in FM control.

Although the loss‐of‐function of the redundant HD‐Zip III genes *PHB* and *PHV*, and the more distantly related *CORONA* (*CNA*) promotes SAM and FM activity [82], we found that both *stm‐2 STTM165/166* and *stm‐2 pPHB:PHBm‐FLAG* flowers with enhanced *PHB* expression produced more floral organs than the wild‐type and *stm‐2* flowers (Figure 3D–F). Consistently, broadened and elevated expression of *PHB* in *phb‐d* mutants results in increased SAM size and ectopic meristem formation [52, 53, 54]. These findings imply a complex role for *PHB* in meristem activity regulation. However, *PHB* was not the key target of SE for pistil restoration in *se‐1 stm‐2*, as pistils were still absent in *stm‐2 STTM165/166* and *stm‐2 pPHB:PHBm‐FLAG* flowers (Figure 3D–F). In addition to the regenerated pistils, ectopic SAMs were also observed in *se‐1 stm‐2* (Figure S2). As previously reported, the loss‐of‐function of *SE* induces a hypertrophic SAM with strong *PHB* expression throughout multiple foci at the flanks of the SAM [9]. Hence, ectopic SAMs in *se‐1 stm‐2* may be a consequence of elevated *PHB* expression (Figure 3C; Figure S2). Furthermore, though we have shown that SE acted through miR171c‐5p, as well as the miR164c‐mediated CUC1/2 pathway to regulate *KNAT2* expression (Figures 4 and 6) and ultimately control FM activity, SE may also respond to *KNOX* expression through the miR165/166‐PHB pathway [9].

In summary, our study demonstrates that SE and STM antagonistically regulate FM activity (Figure 8). While STM promotes both *KNAT2* and *IPT7* expression, SE acts through both miR171c‐5p–KNAT2 and miR164c–CUC1/2–KNAT2 mediated pathways to repress cytokinin signaling, thereby ultimately repressing FM activity. Since FM developmental mechanisms are relatively conserved in flowering species, our work provides insights into FM regulation in crops and horticultural plants. The regulation of pistil architecture and floral density by the SE‐mediated cytokinin pathway holds the potential for crop yield enhancement.

## 4 Experimental Section

### 4.1 Genetic Stocks, Growth Condition, Plant Treatment and Tissue Collection

This study used the *Arabidopsis thaliana* mutants *se‐1*, *stm‐2*, and reporter lines *se‐1 pSE:SE‐eGFP*, *ProTCSn:GFP*, *DR5:GFP‐ER*, *p* *CLV3*:*GFP‐ER*, *pKNAT2:GUS*, and transgenic plants *STTM165/166*, *pPHB:PHBm‐FLAG* and *35S:miR171c*, most of which were in Columbia (Col‐0) ecotype, except that *stm‐2*, *se‐1 stm‐2*, *se‐1 stm‐2 KNAT2‐RNAi*, *STTM171c‐5p*, *STTM164c*, *35S:KNAT2‐GR‐3Myc*, *35S:STM‐GR‐3Myc*, *35S:CUC1‐GR‐3Myc* and *stm‐11 pSTM:STM‐YFP* were in Landsberg erecta (Ler) ecotype. The *stm‐2* mutant was provided by Prof. Xiansheng Zhang and Prof. Yinghua Su (Shandong Agricultural University) and the *pPHB:PHBm‐FLAG* and *STTM165/166* transgenic lines were provided by Prof. Jun Yan (East China Normal University). The mutants of *se‐1* [2], *stm‐2* [83], and *se‐1 stm‐2* [9] were described previously, and the *ProTCSn:GFP* [20], *DR5:GFP‐ER* [41], *pCLV3:GFP‐ER* [84], *stm‐11 pSTM:STM‐YFP* [69], *pPHB:PHBm‐FLAG*, and *STTM165/166* [57, 58] were also previously reported. The *se‐1 pSE:SE‐eGFP*, *35S:KNAT2‐GR‐3Myc*, *35S:STM‐GR‐3Myc*, *35S:CUC1‐GR‐3Myc*, *KNAT2‐RNAi*, *STTM171c‐5p*, *STTM164c*, *35S:miR171c*, and *pKNAT2:GUS* lines were generated in this study. The other double mutants and mutants containing transgenes were generated by genetic crosses and were identified in the F2 generation.

For *se‐1 pSE:SE‐eGFP*, a genomic fragment containing 2241 bp of the promoter upstream of the *SE* start codon and the full‐length genomic DNA (without a stop codon) was fused in‐frame to the *eGFP* sequence and cloned into pCHF1. For *pKNAT2‐GUS* and *pKNAT2‐LUC*, the 1651 and 2469 bp promoters upstream of the *KNAT2* start codon were assembled into MK13‐GUS and pGreenII‐0800‐LUC, respectively. For the *35S:KNAT2‐GR‐3Myc*, *35S:CUC1‐GR‐3Myc* or *35S:STM‐GR‐3Myc* constructs, the CDS of *KNAT2*, *CUC1* or *STM* (without the stop codon) was introduced to the XhoI and ApaI sites of the pGREEN‐II (Invitrogen) vector to express GR‐fused KNAT2, CUC1 or STM protein, all under the control of the Cauliflower Mosaic Virus 35S promoter. For *35S:STM‐eGFP*, *35S:CUC1‐eGFP*, or *35S:KNAT2‐eGFP*, the CDS of *STM*, *CUC1*, or *KNAT2* (without the stop codon), followed by an *eGFP* sequence, was introduced to the MluI and XbaI sites of pGreen‐II. For *35S:miR171c*, the full‐length genomic sequence of *miR171c* was introduced to the MluI and XbaI sites of pGreen‐II. For *KNAT2‐RNAi*, the sequence that is reverse‐complementary to the *KNAT2* coding strand was designed by the Web MicroRNA Designer WMD3 (http://wmd3.weigelworld.org) and cloned into pRS300 according to the manufacturer [85]. The resulting pRS300‐based *KNAT2‐RNAi* cassette was subsequently introduced into the pGreen‐II destination vector.

The *stm‐2* mutant was identified using PCR amplification followed by BccI restriction enzyme digestion, as previously described [80]. All the sequences of the specific primers used are available in Table S1.

DEX (#A601187, BBI) and lovastatin (#A4365, APExBIO) treatments were performed by inverting the plants and submerging the inflorescences in a 10 µM DEX solution or a 10 µM lovastatin solution, respectively, as previously described [86, 87].

Arabidopsis transformation was carried out using the *Agrobacterium tumefaciens* (GV3101) mediated floral dip method, as described previously [88]. Plants with Col‐0 background were backcrossed with Ler at least three times. Arabidopsis and *N. benthamiana* plants were grown in a greenhouse under continuous light at 22°C with 60% relative humidity.

### 4.2 STTM Construction

STTM is a created technique to effectively downregulate a number of miRNAs expressed in various plant species [56]. Taking STTM164c as an example, STTM164c contains two tandem miR164c complementary binding sites separated by a spacer. Each site includes a trinucleotide bulge at the cleavage position, allowing STTM164c to bind miR164c while preventing AGO‐mediated cleavage. This resistance to cleavage ultimately promotes miR164c degradation/sequestration.

*STTM171c‐5p* and *STTM164c* were constructed by following the procedures described previously [55, 56, 57]. In brief, both the *STTM171c‐5p* and *STTM164c* (with trinucleotide mismatches) modules were engineered to form a 96 bp sequence containing the spacer. The STTM modules were first inserted into the SwaI site flanked by the 2×35S promoter and the 35S terminator in pOT2. The recombinant plasmids (∼3.6 kb) were further amplified by a pair of primers (Origin‐del‐PacI) that contained PacI site to delete the plasmid replication origin. The PCR products that contained the STTM and a chloramphenicol selection marker were introduced into a modified pFGC5941 binary vector through the unique PacI site. Recombinant binary plasmids were selected on Luria‐Bertani (LB) plates containing chloramphenicol and kanamycin. The final constructs were verified by DNA sequencing before being used for plant transformation, followed by the selection of positive plants using Basta.

### 4.3 RNA Extraction and RT‐qPCR Analysis

Total RNA was extracted from Arabidopsis inflorescences or *N. benthamiana* leaves using the RNA isolator Total RNA Extraction Reagent Kit (#R711‐01, Vazyme). 2 µg of total RNA was used for reverse transcription by Hifair II first Strand cDNA Synthesis SuperMix for qPCR (#11123, Yeasen) and the assays were performed using the StepOnePlus real‐time PCR system (Applied Biosystems). RT‐qPCR was performed with the gene specific primers listed in Table S1. *ACTIN2* served as the internal reference gene for Arabidopsis.

For the expression of miRNAs, 2 µg of total RNA was used for reverse transcription by the miRNA first Strand cDNA Synthesis Kit (by stem‐loop) (#MR101, Vazyme) with specific miRNA synthesis primers, which were designed following the manufacturer's instructions. The specific miRNA synthesis primers and miRNA‐specific primers for RT‐qPCR are listed in Table S1. All expression assays were carried out for three biological replicates with three technical replicates each.

### 4.4 GO Enrichment Analysis

Gene Ontology (GO) enrichment analysis was performed using the R package clusterProfiler to identify significantly enriched GO terms among the differentially expressed genes between *se‐1 stm‐2* and *stm‐2* [89]. Gene annotation was based on the TAIR10 genome annotation release (The Arabidopsis Information Resource, TAIR), and the generic GO slim ontology was used for functional classification. GO enrichment was calculated using the hypergeometric test implemented in clusterProfiler. P values were adjusted for multiple testing using the Benjamini–Hochberg method. GO terms were ranked according to the false discovery rate (FDR), and the top 15 GO terms with the lowest FDR values were selected for visualization. Enrichment significance was expressed as −log10(FDR‐adjusted *p* value).

### 4.5 Sectioning and Confocal Microscopy Imaging

SAM sections were prepared as previously reported [90]. Briefly, SAM tissues were immersed in a 5% liquid LM agarose solution (#CA1351, Coolaber) at approximately 40°C for embedding and then immediately placed on ice for at least 5 min. Subsequently, the embedded samples were sectioned into 60 µm thick slices using a Leica VT1000S vibratome, and the sections were mounted on microscope slides pre‐coated with a 0.01% MES solution for fluorescence confocal microscopy. During imaging, the GFP and PI channels were acquired. The GFP signal was excited at 488 nm (40% intensity) with an emission range of 493–594 nm and a gain setting of 650, while the PI signal was excited at 405 nm (5% intensity) with an emission range of 410–523 nm and a gain setting of 500.

### 4.6 RNA‐Seq Assays

The RNA‐seq experiment was conducted with two independent biological replicates for each sample. Total RNA (5 µg per sample) of floral buds (≤ stage 8) from *stm‐2* and *se‐1 stm‐2* was used to purify poly (A) mRNA using oligo(dT)‐attached magnetic beads. Library preparation is performed using Optimal Dual‐mode mRNA Library Prep Kit (BGI‐Shenzhen, China). Next, the single‐stranded library products are produced via denaturation. The reaction system for circularization is set up to get the single‐stranded circularized DNA products. Any single stranded linear DNA molecules will be digested. The final single strand circularized library is amplified with phi29 and rolling circle amplification (RCA) to make DNA nanoball (DNB) which carries more than 300 copies of the initial single stranded circularized library molecule. The DNBs are loaded into the patterned nanoarray and sequencing reads with PE 100/150 bases length are generated on G400/T7/T10 platform (BGI‐Shenzhen, China). DNA nanoball (DNB) constitute the fundamental sequencing principle underlying the sequencing platform. The filtration, mapping and subsequent analysis of the sequence reads were performed by G400/T7/T10 platform (BGI‐Shenzhen, China).

Gene expression levels were quantified as transcripts per million (TPM). Genes with zero expression across all samples were removed. The TPM values were log2‐transformed [log2(TPM+1)] and used for principal component analysis (PCA) and sample‐to‐sample Pearson correlation analysis. PCA and correlation heatmap were generated to evaluate the reproducibility among biological replicates. Differential expression was analyzed using DESeq2 and we considered Foldchange > 1.5 and *p* value < 0.05 as differential expression. The number of total and aligned reads obtained for each sample is listed in Dataset S1.

### 4.7 Electrophoretic Mobility Shift Assay (EMSA)

EMSAs were carried out according to a previous procedure [91]. Full length of *STM*, *CUC1*, and *KNAT2* were cloned into the EcoRI site of pGEX4T‐1 and transformed into the *E. coli* strain (Rosetta). GST‐STM, GST‐CUC1, and GST‐KNAT2 were purified by Glutathione resin (#BMR2010, PurKine). Before EMSA, DNA probes were biotin‐labeled and annealed by heating and gradually cooling the double‐stranded DNA to promote proper base pairing of complementary strands. Binding reactions were conducted with an EMSA kit (#20148, Thermo Scientific) to identify interactions between the protein and biotin‐labeled probes. Detailed information regarding the individual probes used is provided in Table S1.

### 4.8 Chromatin Immunoprecipitation (ChIP)

ChIP experiments were performed as described previously [86] with the ChIP Assay Kit (#17‐295, Millipore). Chromatin preparation was performed as described previously [92]. Briefly, inflorescences collected from different plants were ground in liquid nitrogen, postfixed with 1% (w/v) formaldehyde for 10 min, and suspended in M1 buffer (10 mM phosphate buffer, 0.1 M NaCl, 10 mM mercapto‐ethanol, 1 M hexylene glycol, and 0.1 mM PMSF/Protease inhibitor tablet cocktail (TargetMol)). The suspension was filtered through Miracloth (Calbiochem) and centrifuged at 1,000×g for 10 min at 4°C. The pellet, containing crude nuclei, was resuspended in M2 buffer (10 mM phosphate buffer, 0.1 M NaCl, 10 mM mercapto‐ethanol, 1 M hexylene glycol, 10 mM MgCl2, 0.5% Triton‐X, and 0.1 mM PMSF/Protease inhibitor tablet cocktail (TargetMol)) and the suspension was centrifuged at 1,000×g for 10 min. This wash was repeated four times and the last wash was done with M3 buffer (10 mM phosphate buffer, 0.1 M NaCl, 10 mM mercapto‐ethanol, and 0.1 mM PMSF/Protease inhibitor tablet cocktail (TargetMol)). The crude nuclear preparation was suspended with the equal volume of Sonic buffer (10 mM phosphate buffer, 0.1 M NaCl, 0.5% sarkosyl, 10 mM EDTA, and 0.1 mM PMSF/Protease inhibitor tablet cocktail (TargetMol)) and solubilized by sonication (Diagenode bioruptor pico) to generate DNA fragments with an average length of 200–500 bp. The chromatin complex was pre‐cleared with protein‐G agarose beads (#16‐201, Millipore). To estimate the enrichment of STM on *KNAT2* locus, cleared chromatin solution from *stm‐11 pSTM:STM‐YFP* was incubated with GFP‐Trap Agarose (#gta‐20, ChromoTek). To estimate the enrichment of CUC1 on *KNAT2* locus and KNAT2 on *IPT7* locus, cleared chromatin solution from *35S:CUC1‐GR*‐*3Myc* or *35S:KNAT2‐GR*‐*3Myc* was incubated with anti‐Myc antibody (#AE010, ABclonal). The primers used for qPCR analysis are listed in Table S1. For all the ChIP experiments, three biological replicates were performed.

### 4.9 CUT&Tag

CUT&Tag assays were performed with Hyperactive Universal CUT&Tag Assay Kit for Illumina Pro (#TD904, Vazyme) according to manufacturer's recommendations with floral buds (≤ stage 8) collected from *stm‐11 pSTM:STM‐YFP*. Sequencing was then performed by Beijing Genomics Institution‐Shenzhen. We analyzed ChIP‐seq data with ChIP‐Hub computational pipeline [93]. We first removed the potential adapter sequences using the Trimmomatic (v.0.36) [94] with the following parameters: “LEADING:3 TRAILING:3 SLIDINGWINDOW: 4:15 MINLEN:30” (version 0.4.1). Then the trimmed reads were mapped to TAIR10 reference using Bowtie2 (version 2.2.6) with the following parameters “‐q ‐no‐unal ‐sensitive” [95]. The PCR duplicates were removed by Picard tools with the MarkDuplicates function (v2.60, http://broadinstitute.github.io/picard/). We saved properly paired reads with high mapping quality (MAPQ score > 30) for further analysis. We sorted the aligned reads with SAMtools (v1.9) [96] and generated the wiggle tracks by deepTools [97] with the following parameters: “–binSize 10 –normalizeUsing RPKM –smoothLength 60”. To enrich the reads signal around the genes, we used the computeMatrix function and generated the reads distributions with the plotProfile function from the deepTools software with the default parameters. We visualized the generated bigwig files with WashU Epigenome Browser [98] to show the CUT&Tag signal. We use the following parameters for peak calling with MACS2 software [99]: “‐g 107742970 –nomodel –keep‐dup all ‐B –SPMR –call‐summits –shift 50 –extsize 100”. We then used with an Irreproducible Discovery Rate (IDR < 0.05) to select reliable peaks between replicates. For differential peaks identification of STM‐YFP, we used the quantification function in cisDynet [100] to first merge reliable peaks before counting the number of reads within those peaks and then normalizing them using the CPM method. The getDiffPeak function was then used for differential peak identification. We considered |log2FC|≥1 and FDR<0.05 as differential peaks. For the target gene assignment of these differential peaks, we assigned these differential peaks to the nearest TSS with BEDTools [101] closest program. Two biological replicates were prepared and sequenced for each CUT&Tag experiment. The number of total and aligned reads obtained for each sample is listed in Dataset S1.

### 4.10 GUS Staining

GUS staining assays were performed as previously described with slight modifications [102]. The 1651 bp promoter region of *KNAT2* was cloned into the PstI and XbaI sites of *MK13‐GUS* and transformed into Agrobacterium (GV3101‐psoup). The Agrobacteria were co‐infiltrated into leaves of 4‐week‐old tobacco plants and cultured for 48–72 h in the dark. The infiltrated leaves were submerged in GUS staining buffer (15.6 mM NaH_2_PO_4_, 24.4 mM Na_2_HPO_4_, 5 mM K_3_Fe(CN)_6_, 5 mM K_4_Fe(CN)_6_, 10 mM EDTA, 0.1% TritonX‐100, and 0.1% X‐Gluc) and subjected to vacuum infiltration three times for 15 min each to ensure penetration of the staining solution into the leaf tissue. The samples were then incubated at 37°C in the dark overnight and the stained tissues were dehydrated in 70% ethanol for over 24 h. Leaves exhibiting GUS staining were imaged with a Canon (SX60 HS) camera. The GUS staining assays of the SAM in Arabidopsis were performed according to the manufacturer's instructions for the GUS Stain Kit (#G3061, Solarbio).

### 4.11 Dual‐Luciferase Assays

Transient expression assays were performed to determine the activation of *pIPT7:LUC* by KNAT2, and the activation of *pKNAT2:LUC* by CUC1 after 3‐day co‐infiltration in *N. benthamiana* leaves. *pIPT7:LUC* plus *35S:eGFP* and *pKNAT2:LUC* plus *35S:eGFP* were used as the reference. Firefly and Renilla luciferase activities were quantified using Dual‐Luciferase Reporter Assay System (#E1910, Promega). Photos of the fluorescent regions in tobacco leaves were taken by the BIO‐RAD molecular Imager ChemiDocXRS + imaging system. The color gradient indicates different intensities of LUC luminescence. Additionally, RNA was extracted from *N. benthamiana* leaves, and the relative expression of LUC/REN was assessed by RT‐qPCR.

### 4.12 Statistical Analysis

The Pre‐processing of RNA‐seq and CUT&Tag data, which were independently repeated in two biological replicates, were provided in the “RNA‐seq Assays” and “CUT&Tag” sections. Phenotypic data were based on at least 10 plants. RT‐qPCR and ChIP‐qPCR experiments were repeated at least three times. Data are presented as mean ± SD. For different batches of plants, the plants of each batch were grown at the same time in a pairwise fashion for comparison, thus statistical significance was determined by paired Student's *t‐test* (for one variable with two samples), one‐way ANOVA followed by two‐sided Tukey's multiple comparison test (for one variable with more than two samples) and two‐way ANOVA followed by two‐sided Tukey's multiple comparison test (for two variables). A two‐tailed *p < 0.05, **p < 0.01 and ***p < 0.001 were taken to indicate statistical significance. For Figures 3F, 4C, 5D, and 6D, different letters (a, b, c, and d) indicate significant differences between groups (*p < 0.05). The bar plots and statistics were done using GraphPad Prism (v.8; https://www.graphpad.com/features). Detailed results of statistical analysis are available in Dataset S5.

### 4.13 Accession Numbers

Sequence data for genes described in this article can be found in The Arabidopsis Information Resource (TAIR) under the following accession numbers: *SE* (AT2G27100), *STM* (AT1G62360), *WUS* (AT2G17950), *CLV3* (AT2G27250), *KNAT2* (AT1G70510), *BP* (AT4G08150), *KNAT6* (AT1G23380), *CUC1* (AT3G15170), *CUC2* (AT5G53950), *PHB* (AT2G34710), *IPT3* (AT3G63110), *IPT5* (AT5G19040), *IPT7* (AT3G23630), *CKX3* (AT5G56970), *CKX5* (AT1G75450), *LOG1* (AT2G28305), *LOG2* (AT2G35990), *LOG4* (AT3G53450), *ARR1* (AT3G16857), *ARR5* (AT3G48100), *ARR7* (AT1G19050), *ARR10* (AT4G31920), *ARR12* (AT2G25180), *ARR15* (AT1G74890), *ARR18* (AT5G58080), *PIN1* (AT1G73590), *PIN3* (AT1G70940), *PIN4* (AT2G01420), *PIN7* (AT1G23080), *YUC1* (AT4G32540), *YUC2* (AT4G13260), *YUC4* (AT5G11320), *miR171c* (AT1G62035) and *miR164c* (AT5G27807).

## Author Contributions

Conceptualization was carried out by B.S.; methodology by W.Y.; software development by Y.Y., T.Z., and D.C.; investigation by W.Y., W.C., Y.W., Z.W., D.L., X.W., Y.C., and H.Z.; resources were provided by W.Y., Y.W., and Z.W.; the original draft was written by W.Y. and W.C.; review and editing were done by W.C. and B.S.; funding was acquired by W.Y., W.C., and B.S.; project administration was handled by W.C. and B.S.; and supervision was provided by B.S.

## Funding

This work was supported by the National Natural Science Foundation of China (grant 32470358 to B.S. and 32470356 to W.C.), the Research Start‐up Funds for Team Building of Nanjing University (0208/14912231) to W.C., and the Postgraduate Research & Practice Innovation Program of Jiangsu Province (KYCX25_0237) to W.Y.

## Conflicts of Interest

The authors declare no conflicts of interest.

## Supporting information




**Supporting File**: advs75540‐sup‐0001‐SuppMat.pdf.

**Supporting File**: advs75540‐sup‐0002‐Data.zip.

## Data Availability

The raw sequence data reported in this paper have been deposited in the Genome Sequence Archive of the National Genomics Data Center, China National Center for Bioinformation/Beijing Institute of Genomics, Chinese Academy of Sciences (GSA: CRA032747) and are publicly accessible at https://ngdc.cncb.ac.cn/gsa.
