# Supplementary material for: SERRATE Regulates Floral Meristem Activity by Antagonizing SHOOT MERISTEMLESS and Repressing Cytokinin Signaling
Source: Adv Sci (Weinh). 2026 May 7;13(42):e75540. doi: 10.1002/advs.75540 (PMC13335947; doi:10.1002/advs.75540)
Supplement: Supplementary file 1 — Supporting File: advs75540‐sup‐0001‐SuppMat.pdf. [file ADVS-13-e75540-s001.pdf]

Supporting Information

SERRATE regulates floral meristem activity by antagonizing SHOOT MERISTEMLESS and repressing cytokinin signaling

Wen Yang[#], *Yiting Wang*[#], *Yuxin You*, *Zhiyue Wu*, *Dongbao Li*, *Xin Wang*, *Yongsheng Chang*, *Hang Zhao, Tao Zhu*, *Dijun Chen*, *Wei Chen**, *Bo Sun**

W. Yang, Y. Wang, Y. You, Z. Wu, D. Li, X. Wang, Y. Chang, H. Zhao, T. Zhu, D. Chen, W. Chen, B. Sun
State Key Laboratory of Pharmaceutical Biotechnology, School of Life Sciences, Nanjing University, Nanjing, 210023, China
E-mail: cwei@nju.edu.cn; sunbo@nju.edu.cn

This PDF file includes:

Figure S1 to S10

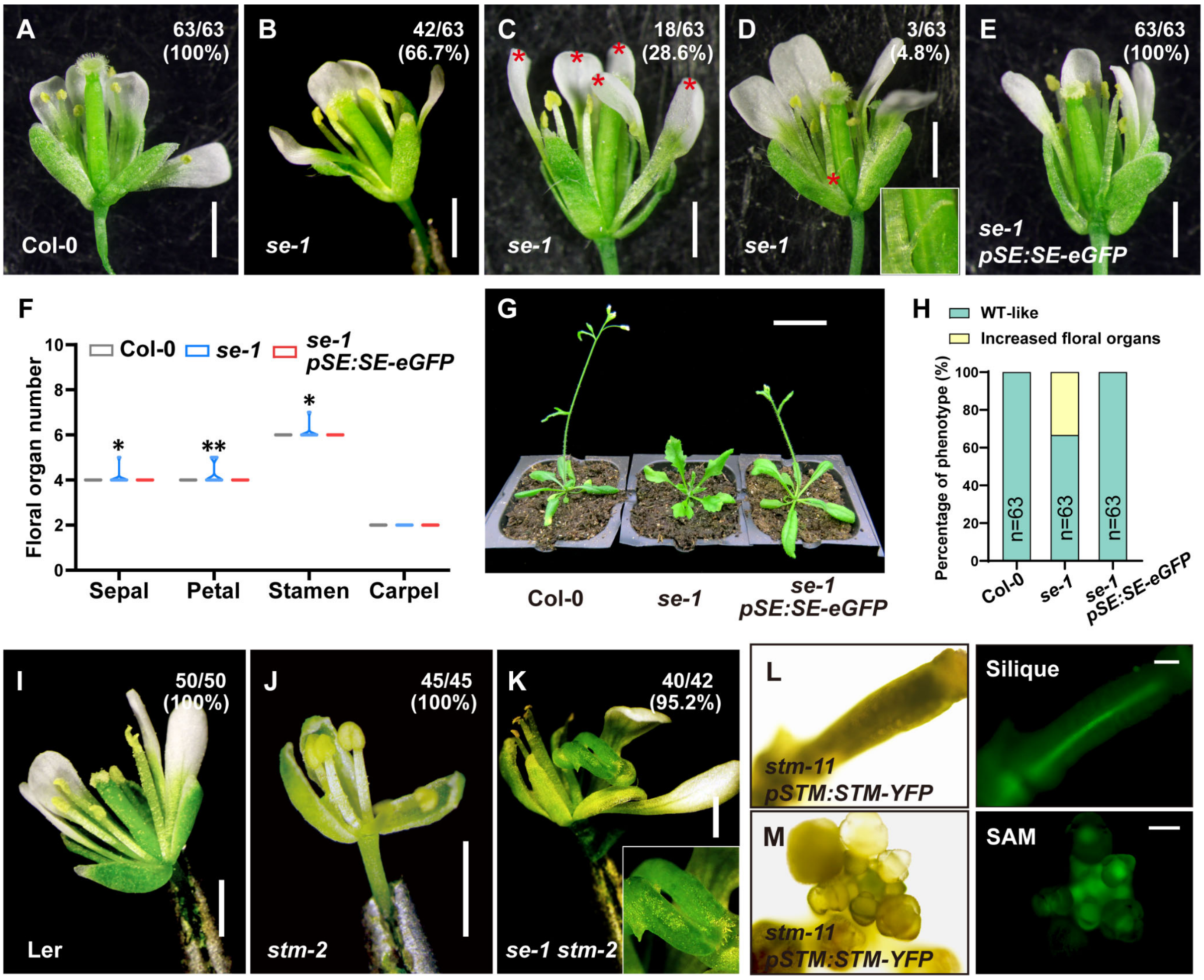

Figure S1. Flower phenotypes of Col-0, *se-1*, *se-1 pSE:SE-eGFP* and STM-YFP signals in *stm-11 pSTM:STM-YFP*. (A-E) Flower phenotypes of Col-0 (A, n=63), *se-1* (B-D, n=63), and *se-1 pSE:SE-eGFP* (E, n=63). Randomly picked flowers from 20 independent plants (3~4 flowers per plant) from three independent repetitions (6~7 plants per repetition) were used for analysis. Red asterisks indicate increased petals (C) and additional filamentous-like structure (D). The insert in (D) is the close-up view of the filamentous-like structure. Scale bars, 1 mm. (F) Statistical analysis of floral organ numbers in Col-0, *se-1*, and *se-1 pSE:SE-eGFP* (n=63). Floral organs of 63 randomly picked flowers from 20 independent plants (3~4 flowers per plant) from three independent repetitions (6~7 plants per repetition) were used for analysis. (G) Phenotype of Col-0, *se-1*, and *se-1 pSE:SE-eGFP* plants. Scale bar, 3 cm. (H) Percentage of flowers with normal (WT-like) and increased numbers of floral organs in Col-0, *se-1,* and *se-1 pSE:SE-eGFP* (n=63). Floral organs of 63 randomly picked flowers from 20 independent plants (3~4 flowers per plant) from three independent repetitions (6~7 plants per repetition) were used for analysis. (I-K) Flower phenotypes of the wild-type (Ler) (I, n=50), *stm-2* (J, n=45), and *se-1 stm-2* (K, n=42). Randomly picked flowers from 20 independent plants (2~3 flowers per plant) from three independent repetitions (6~7 plants per repetition) were used for analysis. The

insert in (K) is the close-up view of the carpel-like structure. Scale bars, 1 mm. (L, M) Observation of STM-YFP signals in the *stm-11 pSTM:STM-YFP* silique (L) and SAM (M). Scale bars, 400 μm. Upper right numbers in (A–E) and (I–K) show the phenotype frequency (individuals with phenotype/total, %). The significant differences are calculated using two-way ANOVA followed by Tukey's multiple comparison test for (F). Statistically significant differences are indicated by **p*<0.05 and ***p*<0.01.

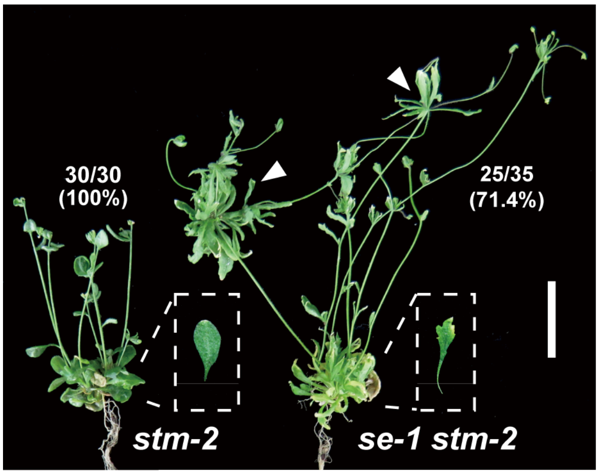

Figure S2. Phenotype of *stm-2* and *se-1 stm-2* plants. The white frames indicate the leaf phenotypes and the white arrowheads indicate the ectopic SAMs in *se-1 stm-2*. The numbers show the phenotype frequency (individuals with phenotype/total, %) and 30 independent plants from each mutant were observed. Scale bar, 3 cm.

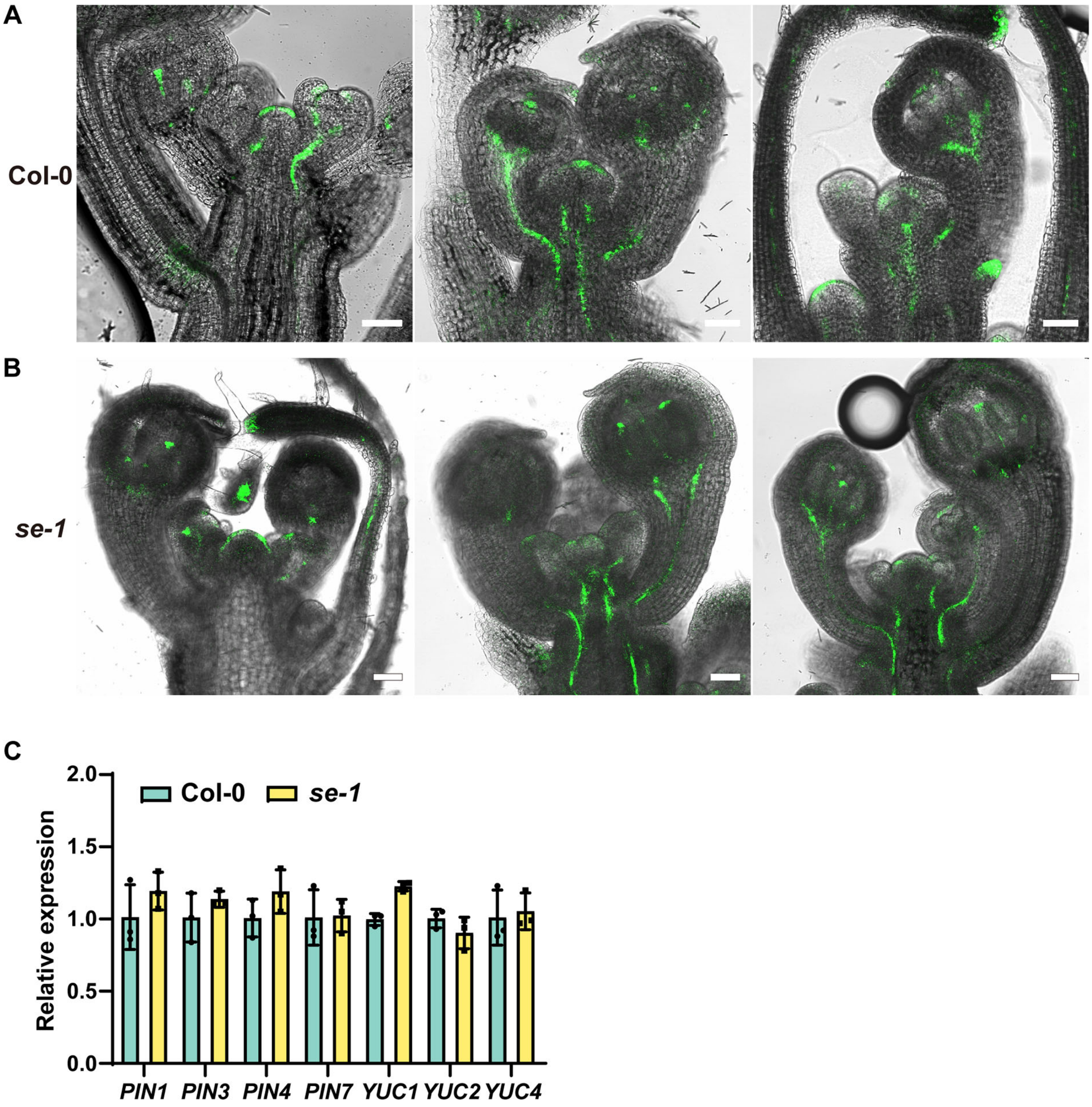

Figure S3. Auxin output in SAM and floral buds of Col-0 and *se-1*. (A, B) Observation of DR5:GFP-ER signals in the SAM and floral buds of Col-0 (A) and *se-1* (B). Scale bar, 50 µm. (C) RT-qPCR analysis of expression levels of auxin-related genes in Col-0 and *se-1* inflorescence. The significant differences are calculated using two-way ANOVA followed by Tukey's multiple comparison test. Independent biological replicates (with three technical replicates each) in (C): n=3. Values are means ± SD.

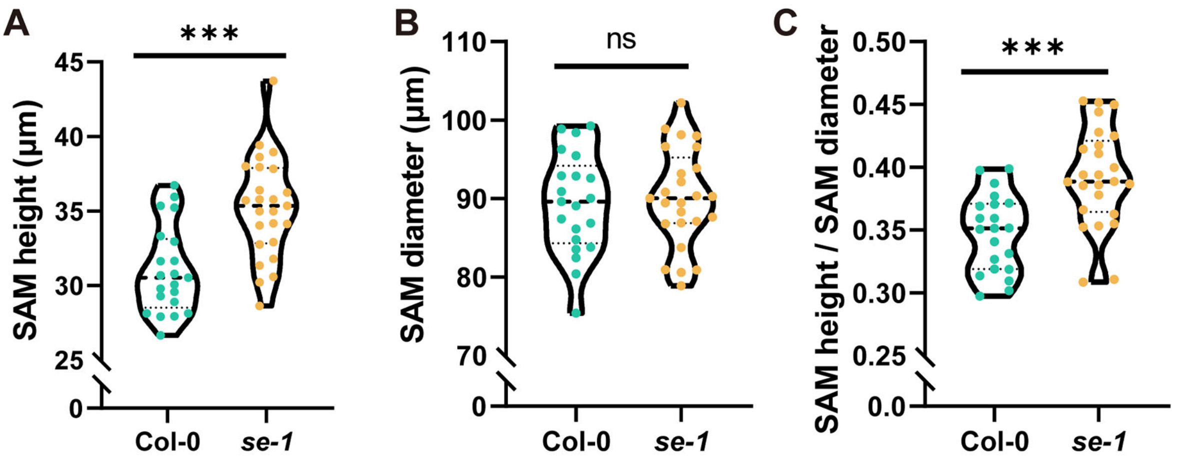

Figure S4. Statistical analysis of SAM heights, SAM diameters, and SAM height/SAM diameter ratios of Col-0 and *se-1*. (A-C) Statistical analysis of SAM heights (A), SAM diameters (B), and SAM height/SAM diameter ratios (C) of Col-0 and *se-1* (n=30). SAMs of 30 randomly picked inflorescences from 30 independent plants (1 inflorescence per plant) from three independent repetitions (10 plants per repetition) were used for analysis. The significant differences are calculated using Student's *t-test*. Statistically significant differences are indicated by $^{**}p<0.01$, $^{***}p<0.001$, and ns (no significant difference).

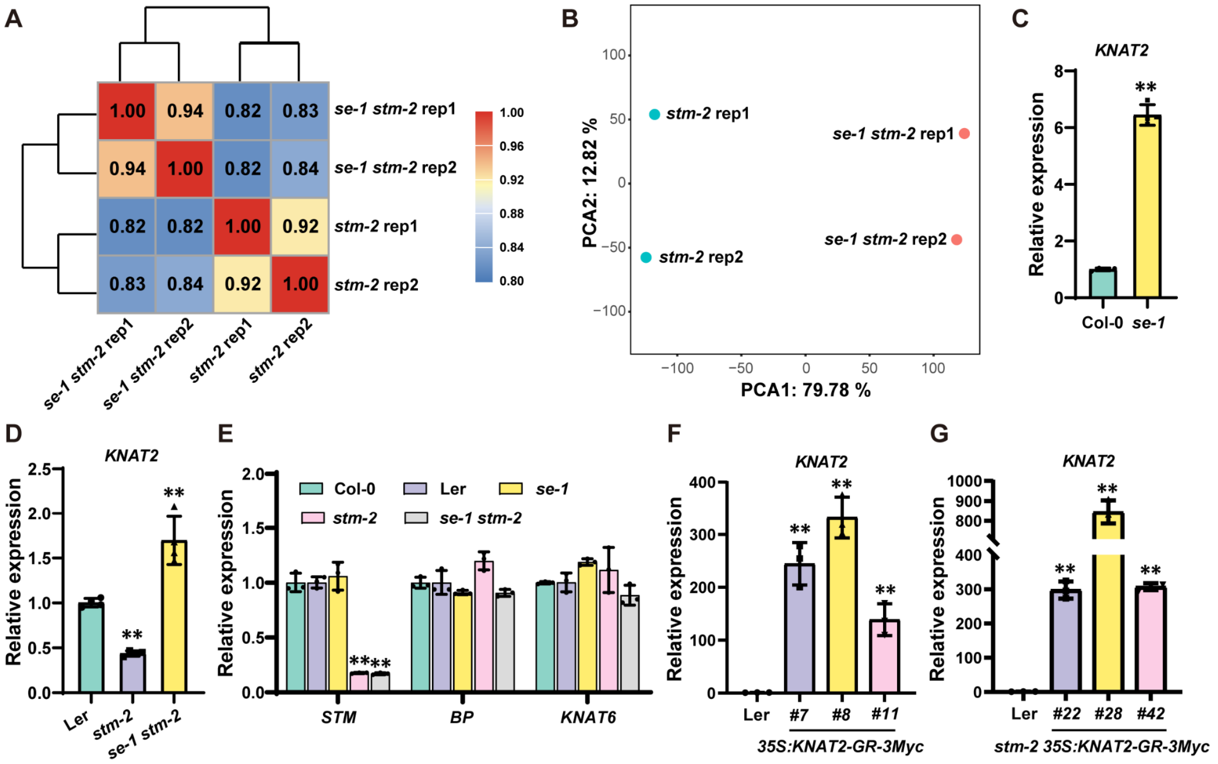

Figure S5. Reproducibility analysis of RNA-seq and RT-qPCR analysis of *KNAT2*, *STM*, *BP*, and *KNAT6* expression levels in different Arabidopsis lines. (A) Pearson correlation coefficients of RNA-seq replicates. (B) Principal component analysis of RNA-seq replicates. (C, D) RT-qPCR analysis of *KNAT2* expression levels in Col-0 and *se-1* (C), and Ler, *stm-2*, and *se-1 stm-2* (D) inflorescence. (E) RT-qPCR analysis of *STM*, *BP*, and *KNAT6* expression levels in Col-0, Ler, *se-1*, *stm-2,* and *se-1 stm-2* inflorescence. (F, G) RT-qPCR analysis of *KNAT2* expression levels in Ler, *35S:KNAT2-GR-3Myc* (F), and *stm-2 35S:KNAT2-GR-3Myc* (G) inflorescence. The significant differences are calculated using Student's *t*-test for (C), one-way ANOVA followed by Tukey's multiple comparison test for (D) and (F-G), and two-way ANOVA followed by Tukey's multiple comparison test for (E). Statistically significant differences are indicated by $^{**}p<0.01$. Independent biological replicates (with three technical replicates each) in (C-G): n=3. Values are means ± SD.

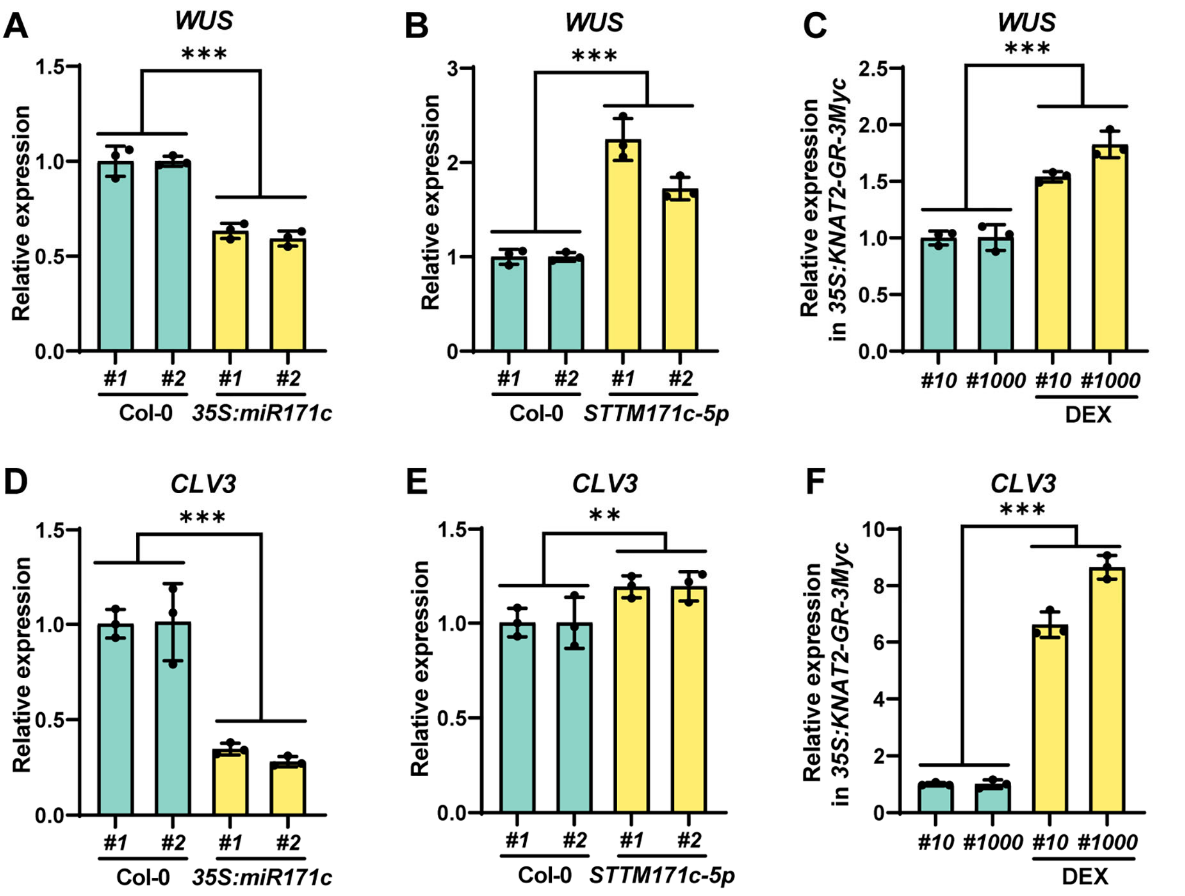

Figure S6. RT-qPCR analysis of *WUS* and *CLV3* expression levels in Col-0, *35S:miR171c*, *STTM171c-5p*, and DEX-induced *35S:KNAT2-GR-3Myc*. (A-C) RT-qPCR analysis of *WUS* expression levels in Col-0, *35S:miR171c* (A), *STTM171c-5p* (B), and DEX induced *35S:KNAT2-GR-3Myc* (C) floral buds. (D-F) RT-qPCR analysis of *CLV3* expression levels in Col-0, *35S:miR171c* (D), *STTM171c-5p* (E), and DEX induced *35S:KNAT2-GR-3Myc* (F) floral buds. The significant differences are calculated using two-way ANOVA followed by Tukey's multiple comparison test. Statistically significant differences are indicated by **p<0.01 and ***p<0.001. Independent biological replicates (with three technical replicates each): n=3. Values are means ± SD.

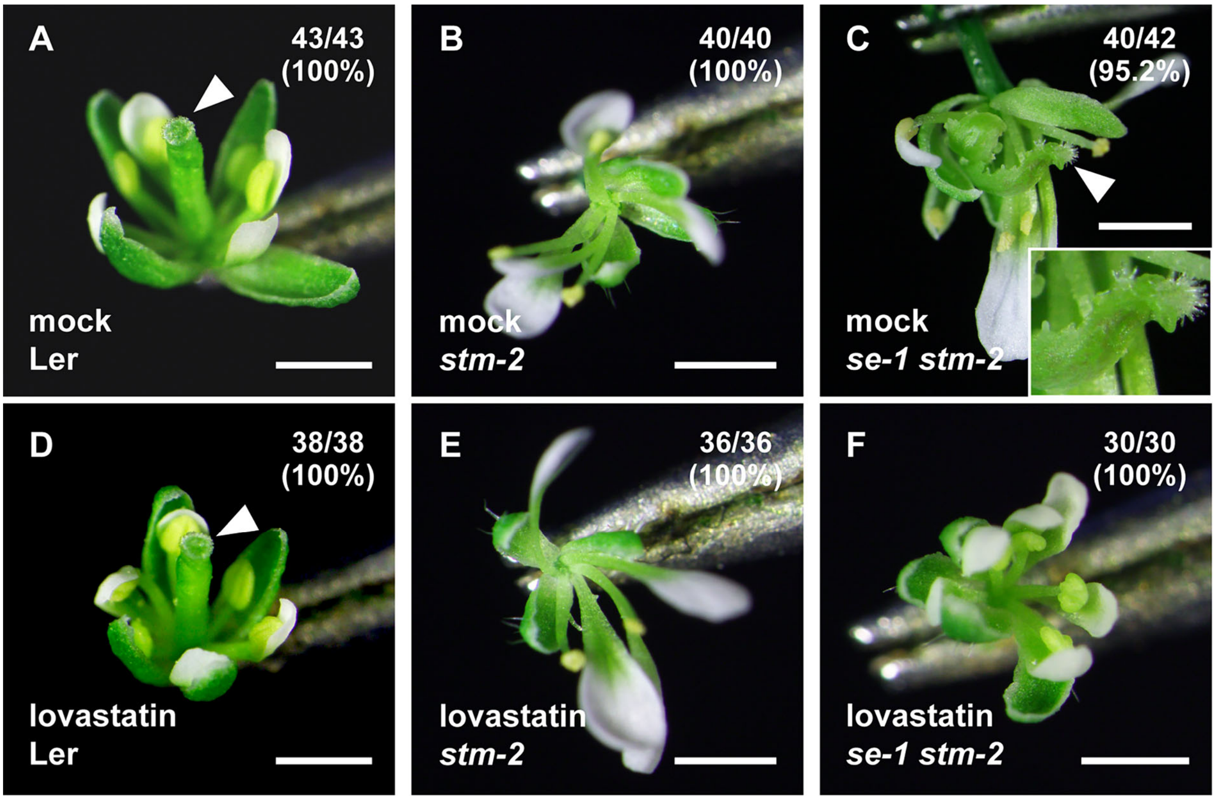

Figure S7. Flower phenotypes of Ler, *stm-2*, and *se-1 stm-2* with or without lovastatin treatment. (A-F) Flower phenotypes of Ler (A, D), *stm-2* (B, E), and *se-1 stm-2* (C, F) after mock (A, B, C) or lovastatin (D, E, F) treatment. Randomly picked flowers from 20 independent plants (1~3 flowers per plant) from two independent repetitions (10 plants per repetition) were used for analysis. The white arrowheads indicate the pistils. The insert in (C) is the close-up view of the carpel-like structure. Upper right numbers in (A–F) show the phenotype frequency (individuals with phenotype/total, %). Scale bars, 1 mm.

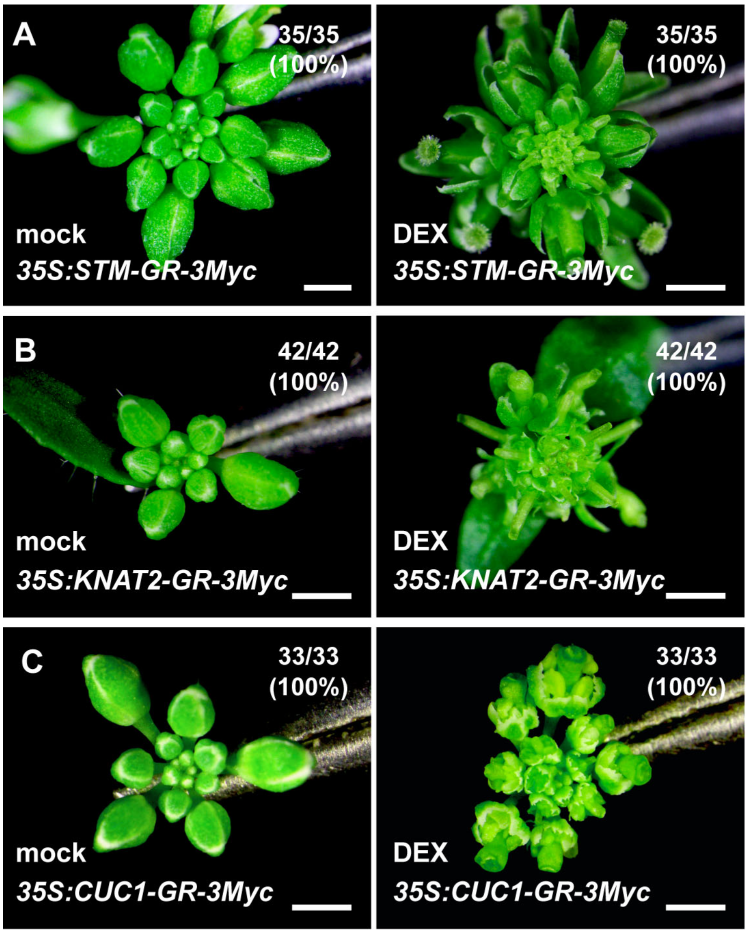

Figure S8. Inflorescence phenotypes of *35S:STM-GR-3Myc*, *35S:KNAT2-GR-3Myc*, and *35S:CUC1-GR-3Myc*. (A-C) Flower phenotypes of DEX-induced (right panels) *35S:STM-GR-3Myc* (A, n=35), *35S:KNAT2-GR-3Myc* (B, n=42), and *35S:CUC1-GR-3Myc* (C, n=33). Randomly picked inflorescences from the same number of independent plants (1 inflorescence per plant) from two independent repetitions (16~21 plants per repetition) were used for analysis. Flower phenotypes of different lines with mock treatment represent the controls (left panels). Upper right numbers in (A–C) show the phenotype frequency (individuals with phenotype/total, %). Scale bars, 1 mm.

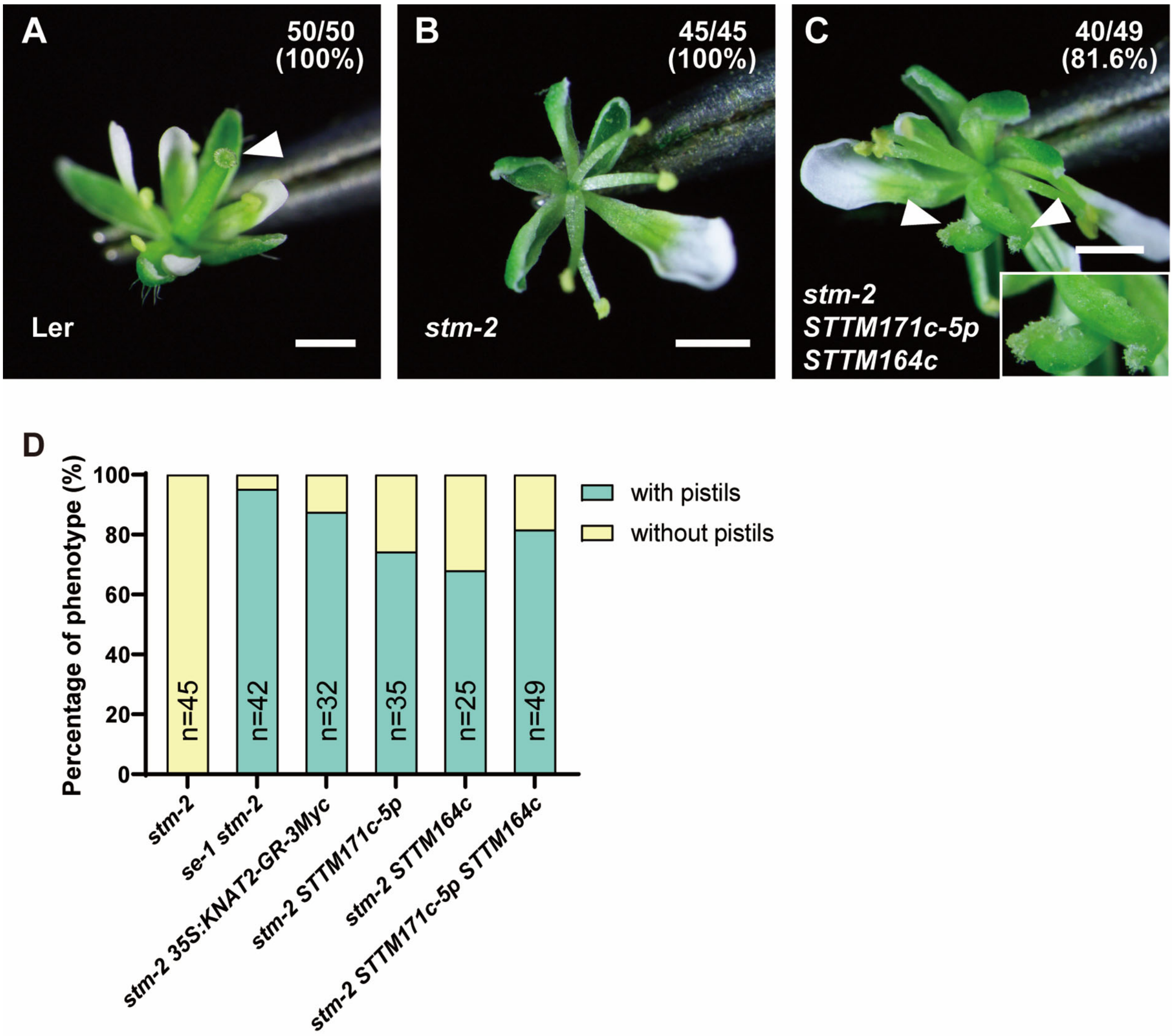

Figure S9. Flower phenotypes of Ler, *stm-2*, and *stm-2 STTM171c-5p STTM164c*. (A-C) Flower phenotypes of Ler (A, n=50), *stm-2* (B, n=45), and *stm-2 STTM171c-5p STTM164c* (C, n=49). Randomly picked flowers from 20 independent plants (2~3 flowers per plant) from two independent repetitions (10 plants per repetition) were used for analysis. The white arrowheads indicate the pistils. The insert in (C) is the close-up view of the carpel-like structure. Scale bars, 1 mm. (D) Percentage of flowers with or without pistils in *stm-2* (n=45), *se-1 stm-2* (n=42), *stm-2 35S:KNAT2-GR-3Myc* (n=32), *stm-2 STTM171c-5p* (n=35), *stm-2 STTM164c* (n=25), and *stm-2 STTM171c-5p STTM164c* (n=49). Randomly picked flowers from 10~20 independent plants (2~3 flowers per plant) from two independent repetitions (5~10 plants per repetition) were used for analysis. Upper right numbers in (A–C) show the phenotype frequency (individuals with phenotype/total, %).

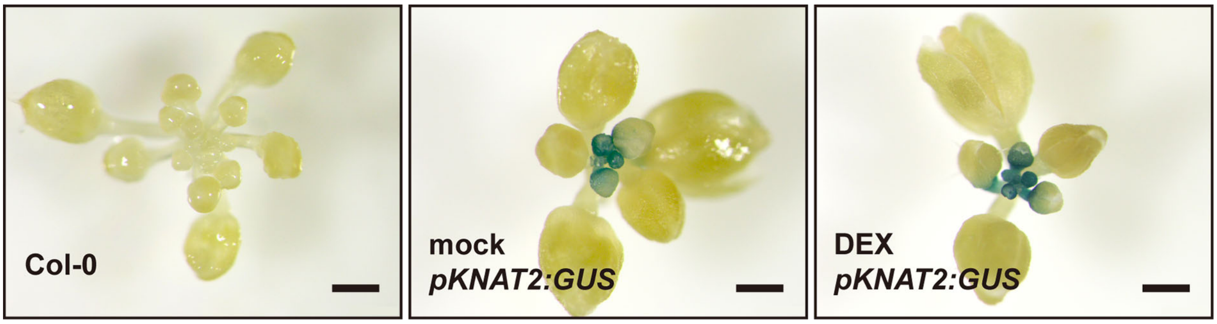

Figure S10. Observation of GUS signals. Observation of GUS signals in Col-0 (left) and *pKNAT2:GUS* inflorescences after mock (middle) and DEX (right) treatments. At least 15 inflorescences of 15 independent plants (1 inflorescence per plant) from two independent repetitions (at least 7~8 plants per repetition) were used for analysis. Scale bars, 500 μm.
